# Efficient Regioselective Synthesis of Novel Condensed Sulfur–Nitrogen Heterocyclic Compounds Based on Annulation Reactions of 2-Quinolinesulfenyl Halides with Alkenes and Cycloalkenes

**DOI:** 10.3390/molecules26164844

**Published:** 2021-08-10

**Authors:** Vladimir A. Potapov, Roman S. Ishigeev, Svetlana V. Amosova

**Affiliations:** A. E. Favorsky Irkutsk Institute of Chemistry, Siberian Division of The Russian Academy of Sciences, 1 Favorsky Str., 664033 Irkutsk, Russia; ishigeev@irioch.irk.ru (R.S.I.); amosova@irioch.irk.ru (S.V.A.)

**Keywords:** 1,2-dihydro[1,3]thiazolo[3,2-*a*]quinolin-10-ium derivatives, annulation reactions, 2-quinolinesulfenyl chloride, 2-quinolinesulfenyl bromide, alkenes, cycloalkenes, 2-mercaptoquinoline

## Abstract

The preparation of novel reagents 2-quinolinesulfenyl chloride and bromide based on available 2-mercaptoquinoline has been described. This approach opens up opportunities for the introduction of 2-quinolinesulfenyl chloride and bromide into organic synthesis. Regioselective synthesis of novel 1,2-dihydro[1,3]thiazolo[3,2-*a*]quinolin-10-ium derivatives in high yields has been developed by annulation reactions of 2-quinolinesulfenyl chloride and bromide with alkenes. Condensed tetracyclic products have been obtained by the reactions of 2-quinolinesulfenyl chloride and bromide with cycloalkenes. The opposite regiochemistry in the reactions with styrene, isoeugenol and 1-alkenes was discussed.

## 1. Introduction

The vast majority of drugs contain heterocyclic fragments in their structures [1]. The sulfur heterocycles are structural parts of many drugs that are used in modern pharmacotherapy [2]. Drugs containing condensed nitrogen and sulfur heterocycles are some of the most commonly used medications [2]. Penicillin and cephalosporin scaffolds contain condensed nitrogen and sulfur heterocycles and represent examples of antibiotics that played an outstanding role in the history and development of pharmaceutical chemistry.

The quinoline derivatives exhibit a variety of biological activities, including antibacterial, antifungal, anticancer, anti-inflammatory, antimalarial and antileishmanial actions [3,4,5,6,7,8,9]. The fluoroquinolone antibiotics (ciprofloxacin, levofloxacin, gatifloxacin and moxifloxacin) and antimalarial medications (chloroquine, hydroxychloroquine, amodiaquine and primaquine) took an important place in pharmacotherapy. It is worth noting that some quinoline derivatives (e.g., hydroxychloroquine) have been recently used for the treatment of COVID-19 [10].

**Figure 1:**
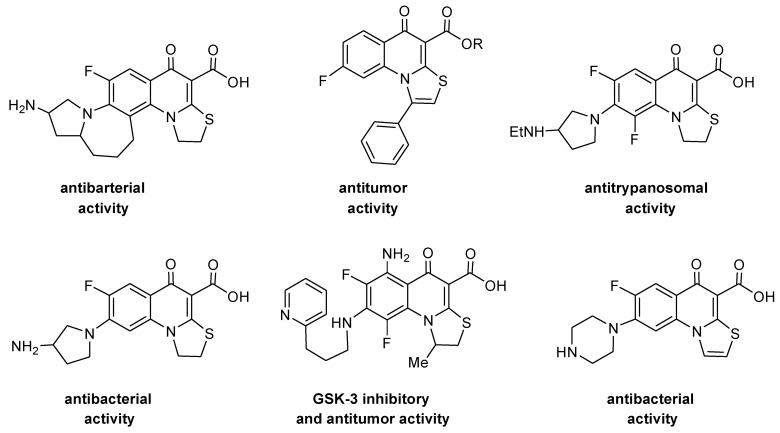
Known biologically active compounds containing [1,3]thiazolo[3,2-*a*]quinolin-10-ium scaffold (antibacterial [11,16,21], antitumor [22,24], antitrypanosomal [26] and glycogen synthase kinase 3 inhibitor [24] activities).

The condensed system of quinoline and thiazole heterocycle is very promising in terms of possible manifestation of biological activity. The [1,3]thiazolo[3,2-*a*]quinolin-10-ium scaffold derivatives represent an important class of condensed heterocyclic compounds exhibiting various types of biological activity including antibacterial [11,12,13,14,15,16,17,18,19,20,21], antitumor [22,23,24], anti-inflammatory [25] and antitrypanosomal [26] action as well as glycogen synthase kinase 3 inhibitors properties [24,27] (Figure 1).

The development of the effective synthesis of novel chalcogen condensed compounds and heterocycles by regioselective annulation and cyclization reactions of chalcogen-containing reagents is our favourable area of research [28,29,30,31,32,33,34,35,36,37,38,39]. The annulation reactions of 2-pyridinesulfenyl and 2-pyridineselenenyl halides with functionalized alkenes led to novel 2*H*,3*H*-[1,3]thiazolo[3,2-*a*]pyridin-4-ium and 2*H*,3*H*-[1,3]selenazolo[3,2-*a*]pyridin-4-ium derivatives in high yields [34,35,36,37,38,39].

**Scheme 1:**
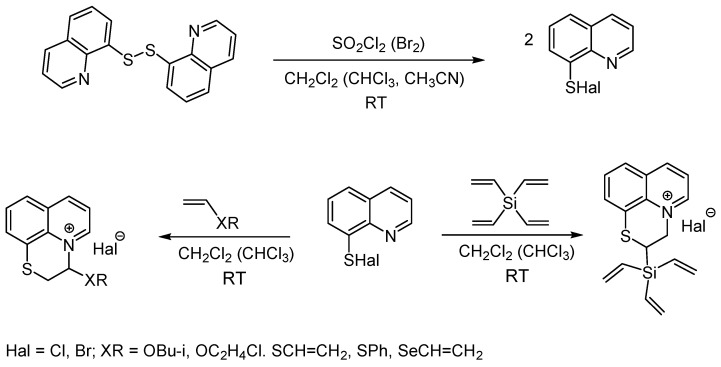
The generation of 8-quinolinesulfenyl halides from di(8-quinolinyl) disulfide by the action of sulfuryl chloride or bromine followed by the annulation reactions with vinylic ethers, sulfides, divinyl selenide and tetravinyl silane.

Recently we described the annulation reactions of 8-quinolinesulfenyl halides with vinylic heteroatom compounds, including vinylic ethers, sulfides, divinyl selenide and tetravinyl silane [40]. Starting 8-quinolinesulfenyl halides were generated in situ from di(8-quinolinyl) disulfide by the action of sulfuryl chloride or bromine and used in further reactions without isolation (Scheme 1).

The annulation reactions of 8-quinolinesulfenyl halides with vinylic ethers, sulfides and divinyl selenide proceeded in a regioselective manner affording 3-substituted 2*H*,3*H*-[1,4]thiazino[2,3,4-*ij*]quinolin-4-ium halides in high yields (Scheme 1) [40]. The annulation reaction of 8-quinolinesulfenyl halides with tetravinyl silane was found to occur with opposite regiochemistry leading to 2-trivinylsilyl-2*H*,3*H*-[1,4]thiazino[2,3,4-*ij*]quinolin-4-ium halides in near quantitative yields (Scheme 1).

**Scheme 2:**
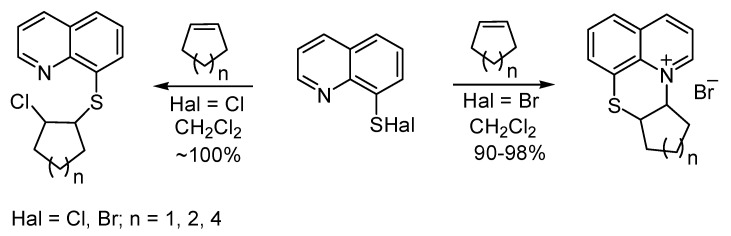
The synthesis of [1,4]thiazino[2,3,4-*ij*]quinolin-11-ium derivatives and 8-[(2-chlorocycloalkyl)sulfanyl]quinolines by the reactions of 8-quinolinesulfenyl chloride and bromide with cycloalkenes.

The reactions of 8-quinolinesulfenyl halides with cycloalkenes (cyclopentene, cyclohexene and cyclooctene), depending on the nature of the halogen, led to [1,4]thiazino[2,3,4-*ij*]quinolin-11-ium derivatives or 8-[(2-chlorocycloalkyl)sulfanyl]quinolines in high yields (90–100%) (Scheme 2). The reactions of 8-quinolinesulfenyl chloride with cycloalkenes led to electrophilic addition products, while condensed compounds were obtained in the case of 8-quinolinesulfenyl bromide (Scheme 2) [41].

The aim of this research is the development of the efficient regioselective synthesis of novel condensed [1,3]thiazolo[3,2-*a*]quinolin-10-ium derivatives with promising biological activity based on annulation reactions of 2-quinolinesulfenyl halides with alkenes and cycloalkenes. The annulation of 5-membered 1,3-thiazole heterocycle to the quinoline ring occurs in the reaction of 2-quinolinesulfenyl halides with alkenes and cycloalkenes, whereas the annulation of 6-membered 1,4-thiazine heterocycle to the quinoline ring is observed in the reactions of 8-quinolinesulfenyl halides with cycloalkenes and vinyl heteroatom compounds [40,41] (Scheme 1 and Scheme 2).

## 2. Results and Discussion

The reactions of 2-quinolinesulfenyl halides are unknown, and the preparation of 2-quinolinesulfenyl chloride and bromide has not yet been described in the literature (the SciFinder database). Previously we generated 8-quinolinesulfenyl halides from di(8-quinolinyl) disulfide by the action of sulfuryl chloride or bromine and involved in further reactions without isolation (Scheme 1). However, di(2-quinolinyl) disulfide is a hard-to-get reagent, whereas 2-mercaptoquinoline is an available compound. It would seem that di(2-quinolinyl) disulfide can be easily prepared by oxidation of 2-mercaptoquinoline. Unfortunately, the oxidation reactions of 2-mercaptoquinoline are complicated by side processes that make it difficult to obtain pure di(2-quinolinyl) disulfide.

**Scheme 3:**
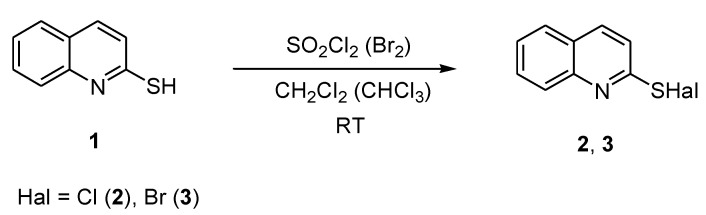
The simple and efficient method for generation of 2-quinolinesulfenyl chloride **2** and bromide **3** by the action of sulfuryl chloride or bromine on 2-mercaptoquinoline **1** in methylene chloride or chloroform.

We developed a simple and efficient method for the generation of 2-quinolinesulfenyl chloride (**2**) and bromide (**3**) by the action of sulfuryl chloride or bromine on 2-mercaptoquinoline (**1**) in methylene chloride or chloroform. The generated 2-quinolinesulfenyl halides **2** and **3** were involved in further reactions without isolation (Scheme 3).

The chemical properties of 2-quinolinesulfenyl chloride **2** and bromide **3** are unknown, and we started studies using simple terminal alkenes (1-hexene, 1-heptene and 1-octene) and cycloalkenes (cyclopentene, cyclohexene and cyclooctene).

**Scheme 4:**
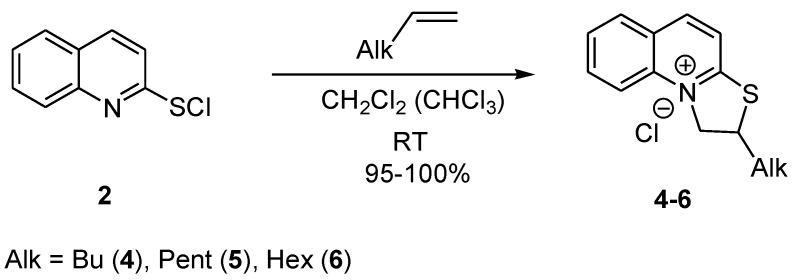
The synthesis of 2-alkyl-1,2-dihydro[1,3]thiazolo[3,2-*a*]quinolin-10-ium chlorides **4**–**6** by the reactions of sulfenyl chloride **2** with 1-hexene, 1-heptene and 1-octene.

We found that the reactions of 2-quinolinesulfenyl chloride **2** with 1-hexene, 1-heptene and 1-octene proceeded in a regioselective fashion at room temperature in methylene chloride or chloroform affording 2-alkyl-1,2-dihydro[1,3]thiazolo[3,2-*a*]quinolin-10-ium chlorides **4**–**6** in 95–100% yields (Scheme 4). The electrophilic addition of the sulfur atom of 2-quinolinesulfenyl chloride **2** occurred to the α-carbon atom of the terminal double bond of alkenes in an anti-Markovnikov fashion.

**Scheme 5:**
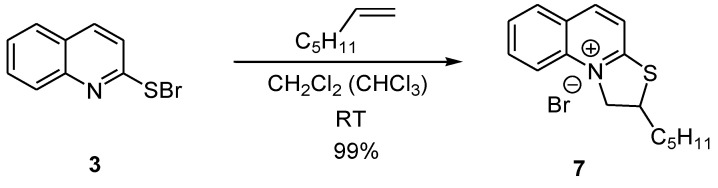
The synthesis of 2-pentyl-1,2-dihydro[1,3]thiazolo[3,2-*a*]quinolin-10-ium bromide **7** by the reactions of sulfenyl bromide **3** with 1-heptene.

Along with the investigation of the chemical properties of sulfenyl chloride **2**, the reactions of 2-quinolinesulfenyl bromide **3** with alkenes were studied. The reaction of sulfenyl bromide **3** with 1-heptene also proceeded regioselectively, leading to condensed compound **7** in a 99% yield (Scheme 5).

**Scheme 6:**
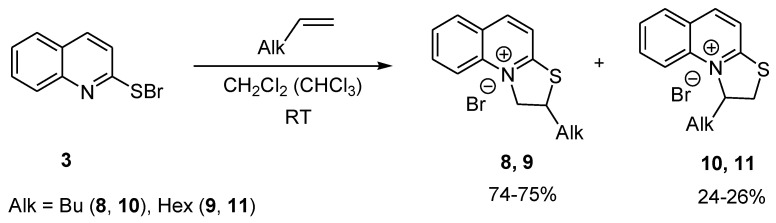
The reactions of 2-quinolinesulfenyl bromide **3** with 1-hexene and 1-octene affording compounds **8**–**11**.

However, the reactions of 2-quinolinesulfenyl bromide **3** with alkenes containing an even number of carbon atoms (1-hexene and 1-octene) occurred with loss of regioselectivity giving along with compounds **8** and **9** (bromide analogues of products **4** and **6**) minor regioisomers **10** and **11**, which were originated from electrophilic addition of the sulfur atom of 2-quinolinesulfenyl bromide **3** to the terminal carbon atom of the double bond of alkenes (Scheme 6). A ratio of regioisomers, compounds **8**/**10** and **9/11**, was found to be approximately 3:1. The total yields of regioisomers **8** + **10** and **9** + **11** are quantitative.

The reactions of 2-quinolinesulfenyl chloride **2** and bromide **3** with cycloalkenes were also studied.

**Scheme 7:**
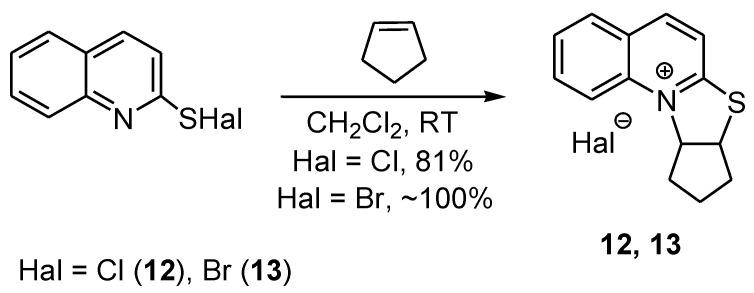
The reactions of 2-quinolinesulfenyl chloride **2** and bromide **3** with cyclopentene leading to condensed tetracyclic compounds **12** and **13**.

Previously we found that the reactions of 8-quinolinesulfenyl bromide with cycloalkenes led to condensed compounds, while in the case of 8-quinolinesulfenyl chloride electrophilic addition, products were obtained (Scheme 2). Unlike this trend, the reactions of cyclopentene with both sulfenyl chloride **2** and bromide **3** at room temperature in methylene chloride afforded condensed tetracyclic products **12** and **13** in 81% and quantitative yields, respectively (Scheme 7).

The reactions of cyclohexene with both 2-quinolinesulfenyl chloride **2** and bromide **3** were accompanied by the formation of by-products, and it was difficult to separate the desired condensed compounds.

**Scheme 8:**
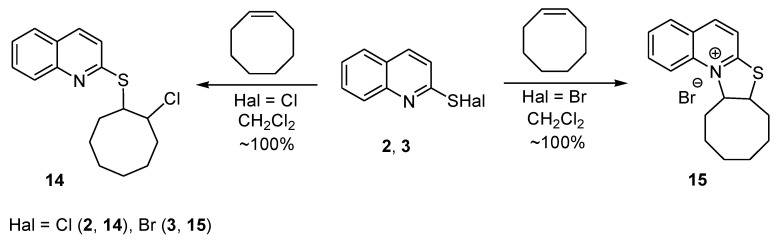
The reactions of 2-quinolinesulfenyl chloride **2** and bromide **3** with cyclooctene affording compounds **14** and **15**.

Unlike the reactions of cyclopentene with both 2-quinolinesulfenyl halides **2** and **3** giving condensed tetracyclic products **12** and **13**, the reaction of 2-quinolinesulfenyl chloride **2** with cyclooctene at room temperature led to electrophilic addition product **14** in quantitative yield (Scheme 8). When sulfenyl bromide **3** was involved in the reaction with cyclooctene under similar conditions, tetracyclic condensed compound **15** was obtained in quantitative yield (Scheme 8).

The formation of an intermediate bromine analogue of compound **14** is accompanied by intramolecular cyclization at room temperature with the formation of a condensed product **15**, while chloro derivative **14** is less reactive under these conditions and remains uncyclized. It is known that the bromine atom is leaving group better than the chlorine atom in nucleophilic substitution reactions, and intramolecular cyclization with the bromine analogue of compound **14** proceeds easier than with chloro derivative **14**.

**Scheme 9:**
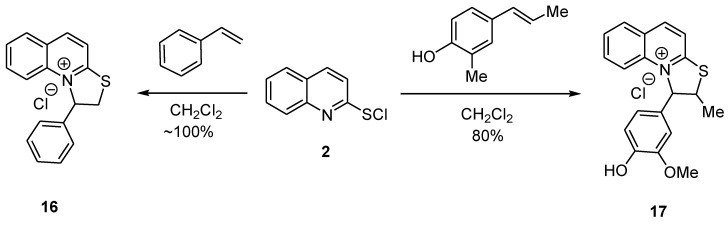
The reactions of 2-quinolinesulfenyl chloride **2** with styrene and isoeugenol giving compounds **16** and **17**.

Finally, we obtained condensed compounds from 2-quinolinesulfenyl chloride **2** and alkenes containing a benzene ring (Scheme 9). The reactions of 2-quinolinesulfenyl chloride **2** with styrene and natural product isoeugenol proceeded at room temperature in methylene chloride in a regioselective manner but with opposite regiochemistry compared to the reactions of sulfenyl chloride **2** with terminal alkenes (Scheme 4). The electrophilic addition of the sulfur atom of sulfenyl chloride **2** occurred to the β-carbon atom of the double bond of styrene and isoeugenol in a Markovnikov fashion. Condensed products **16** and **17** based on styrene and isoeugenol were obtained in quantitative and 80% yields, respectively (Scheme 9).

**Scheme 10:**
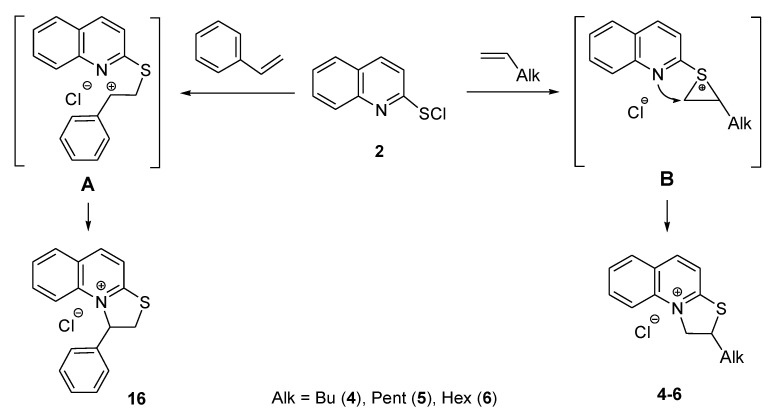
The supposed pathways of the reactions of sulfenyl chloride **2** with styrene and 1-alkenes.

Why do annulation reactions of quinolinesulfenyl chloride **2** with styrene, isoeugenol and terminal alkenes proceed with opposite regiochemistry? Supposed reaction pathways can be regarded in order to explain this trend (Scheme 10). The reactions of sulfenyl chloride **2** with compounds containing a double bond conjugated with the benzene ring (styrene, isoeugenol) proceeded regioselectively via electrophilic addition of the sulfur atom to the β-carbon atom of the double bond. The regioselectivity is due to the formation of intermediate linear carbocation **A**, which is stabilized by the benzene ring (the relatively stable benzyl cation) (Scheme 10).

Addition reactions of sulfenyl halides to alkenes are well understood [42,43,44,45,46,47,48,49,50,51,52,53], and it is known that the reactions of arylsulfenyl halides with styrene and its derivatives also afforded Markovnikov adducts [42,43].

It is worth noting that electrophilic addition of arylsulfenyl halides to linear 1-alkene afforded predominantly anti-Markovnikov products, and thiiranium cations were regarded as intermediates in these reactions [44,45,46,47]. Taking into account these data, we suppose that the reactions of quinolinesulfenyl chloride **2** with terminal alkenes proceeded via intermediates thiiranium cation **B** and nucleophilic attack of the nitrogen atom of the quinoline ring occurred at the least substituted carbon atom of thiiranium ion **B** leading to the formation of products **4**–**6** in an anti-Markovnikov fashion (Scheme 10).

The structural assignments of synthesized compounds were made using ^1^H and ^13^C-NMR spectroscopy (see Supplementary Materials), including proton-coupled ^13^C-NMR spectra, and confirmed by elemental analysis.

The products of opposite regiochemistry exhibit characteristic signals of the carbon atoms bonded with charged nitrogen (N^+^), sulfur atom and with one or two protons in ^13^C-NMR spectra of the obtained compounds (the number of protons is determined by NMR experiments). The CHS moiety and the CH_2_N^+^ methylene group manifest themselves in the regions of 44–48 ppm and 58–62 ppm, respectively, in ^13^C-NMR spectra of compounds **4**–**9** (the products derived from anti-Markovnikov addition of the sulfur electrophile to the double bond). Signals of the CH_2_S group and containing one proton CHN^+^ moiety are observed in the regions of 31–38 ppm and 66–71 ppm, respectively, in ^13^C-NMR spectra of compounds **10**, **11** and **16** (the products derived from Markovnikov addition of the sulfur electrophile to the double bond).

Stereochemistry of compound **17** is determined based on the value of the spin–spin coupling constant (^3^*J*_H-H_ = 8.2 Hz) of protons in the N-(Ar)CH-CH(Me)-S fragment. This value corresponds to the *trans*-disposition of methyl and aryl groups relative to the plane of the ring (*trans*-configuration) [39,54,55].

## 3. Experimental Section

### 3.1. General Information

^1^H (400.1 MHz) and ^13^C (100.6 MHz) NMR spectra were recorded on a Bruker DPX-400 spectrometer (Bruker BioSpin GmbH, Rheinstetten, Germany) in 2–5% solution in D_2_O. ^1^H and ^13^C chemical shifts (δ) are reported in parts per million (ppm), relative to tetramethylsilane (external) or to the residual solvent peaks of D_2_O (δ = 4.79), acetone-*d*_6_ (δ = 2.05 and 29.84 ppm) and DMSO-*d*_6_ (δ = 2.50 and 39.52 ppm for ^1^H and ^13^C NMR, respectively). Elemental analysis was performed on a Thermo Scientific FLASH 2000 Organic Elemental Analyzer (Thermo Fisher Scientific Inc., Milan, Italy). Melting points were determined on a Kofler Hot-Stage Microscope PolyTherm A apparatus (Wagner & Munz GmbH, München, Germany). Absolute solvents were used in the reactions.

### 3.2. Synthesis of Compounds ***4**–**11*** by the Reactions of 2-Quinolinesulfenyl Chloride ***2*** and Bromide ***3*** with Alkenes

*2-Butyl-1,2-dihydro[1,3]thiazolo[3,2-a]quinolin-10-ium chloride* (**4**). A solution of sulfuryl chloride (0.074 g, 0.55 mmol) in methylene chloride (5 mL) was added dropwise to a solution of 2-mercaptoquinoline (0.089 g, 0.55 mmol) in methylene chloride (5 mL), and the mixture was stirred for 15 min at room temperature. The obtained solution of 2-quinolinesulfenyl chloride was added dropwise to a solution of 1-hexene (0.093 g, 1.1 mmol) in methylene chloride (5 mL), and the reaction mixture was stirred for 20 h at room temperature. The solvent was removed by a rotary evaporator. The residue was washed with CCl_4_ and dried in vacuum, giving product **4** (0.146 g, 95% yield) as a yellow oil.

^1^H–NMR (400 MHz, D_2_O): δ 0.81 (t, *J* 7.1 Hz, 3H, CH_3_), 1.25–1.40 (m, 4H, CH_2_), 1.77–1.88 (m, 1H, CH_2_), 1.89–1.99 (m, 1H, CH_2_), 3.79 (tt, *J* 8.7, 6.4 Hz, 1H, SCH,), 5.00 (dd, *J* 13.0, 6.4 Hz, 1H, NCH_2_), 5.25 (dd, *J* 13.0, 8.7 Hz, 1H, NCH_2_), 7.66–7.72 (m, 2H, C_quino_), 7.86–7.88 (m, 1H, C_quino_), 7.93–7.98 (m, 1H, C_quino_), 8.00–8.02 (m, 1H, C_quino_), 8.50–8.52 (m, 1H, C_quino_).

^13^C–NMR (101 MHz, D_2_O): δ 11.82 (CH_3_), 20.27, 27.17, 32.72 (CH_2_), 45.44 (SCH), 59.82 (NCH_2_), 116.27 (C_quino_), 116.84 (C_quino_), 124.96 (C_quino_), 127.06 (C_quino_), 128.85 (C_quino_), 133.87 (C_quino_), 136.51 (C_quino_), 143.49 (C_quino_), 163.60 (C_quino_).

Anal. Calcd for C_15_H_18_NClS: C 64.38, H 6.48, Cl 12.67, N 5.01, S 11.46. Found: C 64.67, H 6.71, Cl 12.00, N 5.29, S 11.89.

*2-Pentyl-1,2-dihydro[1,3]thiazolo[3,2-a]quinolin-10-ium chloride* (**5**). A solution of sulfuryl chloride (0.062 g, 0.46 mmol) in methylene chloride (5 mL) was added dropwise to a solution of 2-mercaptoquinoline (0.074 g, 0.46 mmol) in methylene chloride (5 mL), and the mixture was stirred for 15 min at room temperature. The obtained solution of 2-quinolinesulfenyl chloride was added dropwise to a solution of 1-heptene (0.09 g, 0.92 mmol) in methylene chloride (5 mL), and the reaction mixture was stirred for 20 h at room temperature. The solvent was removed by a rotary evaporator. The residue was washed with CCl_4_ and dried in vacuum, giving product **5** (0.120 g, 100% yield) as a yellow oil.

^1^H–NMR (400 MHz, D_2_O): δ 0.79–0.82 (m, 3H, CH_3_), 1.22–1.30 (m, 4H, CH_2_), 1.37–1.46 (m, 2H, CH_2_), 1.80–1.89 (m, 1H, CH_2_), 1.90–1.97 (m, 1H, CH_2_), 4.49 (ddd, *J* 14.8, 8.7, 6.2 Hz, 1H, SCH), 5.05 (dd, *J* 13.0, 6.2 Hz, 1H, NCH_2_), 5.30 (dd, *J* 13.0, 8.7 Hz, 1H, NCH_2_), 7.72–7.77 (m, 2H, C_quino_), 7.91–7.93 (m, 1H, C_quino_), 7.98–8.3 (m, 1H, C_quino_), 8.06–8.08 (m, 1H, C_quino_), 8.56–8.58 (m, 1H, C_quino_).

^13^C–NMR (101 MHz, D_2_O): δ 11.92 (CH_3_), 20.44, 24.66, 29.18, 32.99 (CH_2_), 45.46 (SCH), 59.82 (NCH_2_), 116.27 (C_quino_), 116.85 (C_quino_), 125.03 (C_quino_), 127.07 (C_quino_), 128.89 (C_quino_), 133.88 (C_quino_), 136.59 (C_quino_), 143.54 (C_quino_), 163.60 (C_quino_).

Anal. Calcd for C_16_H_20_NClS: C 65.40, H 6.86, Cl 12.06, N 4.77, S 10.91. Found: C 65.57, H 7.02, Cl 12.57, N 4.93, S 11.42.

*2*-*Hexyl-1,2-dihydro[1,3]thiazolo[3,2-a]quinolin-10-ium chloride* (**6**). A solution of sulfuryl chloride (0.052 g, 0.39 mmol) in methylene chloride (5 mL) was added dropwise to a solution of 2-mercaptoquinoline (0.063 g, 0.39 mmol) in methylene chloride (5 mL), and the mixture was stirred for 15 min at room temperature. The obtained solution of 2-quinolinesulfenyl chloride was added dropwise to a solution of 1-octene (0.096 g, 0.78 mmol) in methylene chloride (5 mL), and the reaction mixture was stirred for 20 h at room temperature. The solvent was removed by a rotary evaporator. The residue was washed with CCl_4_ and dried in vacuum, giving product **6** (0.097 g, 100% yield) as a light-yellow oil.

^1^H–NMR (400 MHz, D_2_O): δ 0.79–0.82 (m, 3H, CH_3_), 1.21–1.24 (m, 4H, CH_2_), 1.29–1.32 (m, 2H, CH_2_), 1.40–1.45 (m, 2H, CH_2_), 1.82–1.89 (m, 1H, CH_2_), 1.92–1.99 (m, 1H, CH_2_), 4.49–4.53 (m, 1H, SCH), 5.00 (dd, *J* 13.0, 6.3 Hz, 1H, NCH_2_), 5.25 (dd, *J* 13.0, 8.7 Hz, 1H, NCH_2_), 7.74–7.79 (m, 2H, C_quino_), 7.94–7.96 (m, 1H, C_quino_), 8.00–8.03 (m, 1H, C_quino_), 8.09–8.11 (m, 1H, C_quino_), 8.59–8.61 (m, 1H, C_quino_).

^13^C–NMR (101 MHz, D_2_O): δ 12.03 (CH_3_), 20.55, 24.90, 26.60, 29.49, 33.03 (CH_2_), 45.47 (SCH), 59.86 (NCH_2_), 116.31 (C_quino_), 116.87 (C_quino_), 124.98 (C_quino_), 126.61 (C_quino_), 128.94 (C_quino_), 133.79 (C_quino_), 137.84 (C_quino_), 143.59 (C_quino_), 165.00 (C_quino_).

Anal. Calcd for C_17_H_22_NClS: C 66.32, H 7.20, Cl 11.52, N 4.55, S 10.42. Found: C 66.58, H 7.39, Cl 11.88, N 4.72, S 10.90.

*2-Pentyl-1,2-dihydro[1,3]thiazolo[3,2-a]quinolin-10-ium bromide* (**7**). A solution of bromide (0.055 g, 0.34 mmol) in methylene chloride (5 mL) was added dropwise to a solution of 2-mercaptoquinoline (0.055 g, 0.34 mmol) in methylene chloride (5 mL), and the mixture was stirred for 15 min at room temperature. The obtained solution of 2-quinolinesulfenyl bromide was added dropwise to a solution of 1-heptene (0.067 g, 0.68 mmol) in methylene chloride (5 mL), and the reaction mixture was stirred for 20 h at room temperature. The solvent was removed by a rotary evaporator. The residue was washed with CCl_4_ and dried in vacuum, giving product **7** (0.114 g, 99% yield) as an orange oil.

^1^H–NMR (400 MHz, D_2_O): δ 0.73 (t, *J* 6.8 Hz, 3H, CH_3_,), 1.15–1.24 (m, 4H, CH_2_), 1.30–1.42 (m, 2H, CH_2_), 1.77–1.92 (m, 2H, CH_2_), 4.40–4.47 (m, 1H, SCH), 5.02 (dd, *J* 12.9, 6.3 Hz, 1H, NCH_2_), 7.34 (d, *J* 12.9, 8.8 Hz, 1H, NCH_2_), 7.65–7.71 (m, 2H, C_quino_), 7.86–7.89 (m, 1H, C_quino_), 7.92–7.96 (m, 1H, C_quino_), 8.01–8.03 (m, 1H, C_quino_), 8.51–8.53 (m, 1H, C_quino_).

^13^C–NMR (101 MHz, D_2_O): δ 14.14 (CH_3_), 22.65, 26.89, 31.38, 35.20 (CH_2_), 47.67 (SCH), 62.09 (NCH_2_), 118.56 (C_quino_), 119.56 (C_quino_), 127.30 (C_quino_), 129.20 (C_quino_), 131.09 (C_quino_), 136.00 (C_quino_), 145.72 (C_quino_).

Anal. Calcd for C_16_H_20_NBrS: C 56.80, H 5.96, Br 23.62, N 4.14, S 9.48. Found: C 57.02, H 6.21, Br 23.98, N 4.40, S 9.91.

*2-Butyl-1,2-dihydro[1,3]thiazolo[3,2-α]quinolin-10-ium bromide* (**8**) and *1-butyl-1,2-dihydro[1,3]thiazolo[3,2-α]quinolin-10-ium bromide* (**10**). A solution of bromide (0.048 g, 0.30 mmol) in methylene chloride (5 mL) was added dropwise to a solution of 2-mercaptoquinoline (0.048 g, 0.30 mmol) in methylene chloride (5 mL), and the mixture was stirred for 15 min at room temperature. The obtained solution of 2-quinolinesulfenyl bromide was added dropwise to a solution of 1-hexene (0.050 g, 0.60 mmol) in methylene chloride (5 mL), and the reaction mixture was stirred for 20 h at room temperature. The solvent was removed by a rotary evaporator. The residue was washed with CCl_4_ and dried in vacuum, giving a mixture (0.097 g) of products **8** (0.073 g, 75% yield) and **10** (0.024 g, 24% yield) as a yellow oil.

*2-Butyl-1,2-dihydro[1,3]thiazolo[3,2-a]quinolin-10-ium bromide* (**8**). ^1^H–NMR (400 MHz, D_2_O): δ 0.80–0.87 (m, 3H, CH_3_), 1.28–1.52 (m, 4H, CH_2_), 1.83–1.92 (m, 1H, CH_2_), 1.94–2.01 (m, 1H, CH_2_), 4.52 (dd, *J* 8.3, 6.4 Hz, 1H, SCH), 5.05 (dd, *J* 13.0, 6.4 Hz, 1H, NCH_2_), 5.30 (dd, *J* 13.0, 8.3 Hz, 1H, NCH_2_), 7.71–7.79 (m, 2H, C_quino_), 7.91–7.93 (m, 1H, C_quino_), 7.97–8.02 (m, 1H, C_quino_), 8.05–8.07 (m, 1H, C_quino_), 8.54–8.56 (m, 1H, C_quino_).

^13^C–NMR (101 MHz, D_2_O) (multiplicities are given based on the proton-coupled ^13^C-NMR spectrum): δ 10.36 (q, CH_3_), 18.80, 25.69, 31.25 (t, CH_2_), 43.97 (d, SCH), 58.35 (t, NCH_2_), 114.80 (d, C_quino_), 115.37 (d, C_quino_), 123.40 (d, C_quino_), 125.58 (s, C_quino_), 127.34 (d, C_quino_), 132.40 (d, C_quino_), 134.97 (s, C_quino_), 141.99 (d, C_quino_), 162.08 (s, C_quino_).

*1-Butyl-1,2-dihydro[1,3]thiazolo[3,2-a]quinolin-10-ium bromide* (**10**). ^1^H–NMR (400 MHz, D_2_O): δ 0.80–0.87 (m, 3H, CH_3_), 1.28–1.52 (m, 4H, CH_2_), 1.58–1.67 (m, 1H, CH_2_), 1.94–2.01 (m, 1H, CH_2_), 3.79 (d, *J* 12.1 Hz, 1H, SCH_2_), 5.02 (dd, *J* 12.1, 8.3 Hz, 1H, SCH_2_), 5.96 (t, *J* 9.4 Hz, 1H, NCH), 7.71–7.79 (m, 2H, C_quino_), 8.00–8.04 (m, 2H, C_quino_), 8.05–8.07 (m, 1H, C_quino_), 8.54–8.56 (m, 1H, C_quino_).

^13^C–NMR (101 MHz, D_2_O) (multiplicities are given based on the proton-coupled ^13^C-NMR spectrum): δ 10.36 (q, CH_3_), 18.95, 24.13, 26.85 (t, CH_2_), 30.99 (t, SCH_2_), 66.61 (d, NCH), 115.10 (d, C_quino_), 115.55 (d, C_quino_), 123.42 (d, C_quino_), 125.60 (s, C_quino_), 127.67 (d, C_quino_), 132.39 (d, C_quino_), 134.98 (s, C_quino_), 141.97 (d, C_quino_), 161.66 (s, C_quino_).

Anal. Calcd for C_15_H_18_NBrS: C 55.56, H 5.59, Br 24.64, N 4.32, S 9.89. Found: C 55.89, H 5.78, Br 24.91, N 4.60, S 10.25.

*2-Hexyl-1,2-dihydro[1,3]thiazolo[3,2-a]quinolin-10-ium bromide* (**9**) and *1-hexyl-1,2-dihydro[1,3]thiazolo[3,2-a]quinolin-10-ium bromide* (**11**). A solution of bromide (0.063 g, 0.39 mmol) in methylene chloride (5 mL) was added dropwise to a solution of 2-mercaptoquinoline (0.063 g, 0.39 mmol) in methylene chloride (5 mL), and the mixture was stirred for 15 min at room temperature. The obtained solution of 2-quinolinesulfenyl bromide was added dropwise to a solution of 1-octene (0.096 g, 0.78 mmol) in methylene chloride (5 mL), and the reaction mixture was stirred for 20 h at room temperature. The solvent was removed by a rotary evaporator. The residue was washed with CCl_4_ and dried in vacuum, giving a mixture (0.12 g) of products **9** (0.089 g, 74% yield) and **11** (0.031 g, 26% yield) as a yellow oil.

*2-Hexyl-1,2-dihydro[1,3]thiazolo[3,2-a]quinolin-10-ium bromide* (**9**). ^1^H–NMR (400 MHz, DMSO-*d*_6_): δ 0.80–0.84 (m, 3H, CH_3_), 1.26–1.32 (m, 6H, CH_2_), 1.36–1.51 (m, 2H, CH_2_), 1.84–1.99 (m, 2H, CH_2_), 4.59–4.63 (m, 1H, SCH), 5.30–5.34 (m, 1H, NCH_2_), 5.45–5.49 (m, 1H, NCH_2_), 7.85–7.89 (m, 1H, C_quino_), 8.12–8.15 (m, 1H, C_quino_), 8.20–8.25 (m, 1H, C_quino_), 8.28–8.30 (m, 1H, C_quino_), 8.32–8.37 (m, 1H, C_quino_), 8.96–8.98 (m, 1H, C_quino_).

^13^C–NMR (101 MHz, DMSO-*d*_6_): δ 13.93 (CH_3_), 21.99, 26.40, 28.23, 31.02, 34.21 (CH_2_), 46.57 (SCH), 61.17 (NCH_2_), 118.80 (C_quino_), 119.03 (C_quino_), 126.28 (C_quino_), 128.16 (C_quino_), 130.06 (C_quino_), 134.78 (C_quino_), 137.90 (C_quino_), 144.62 (C_quino_), 164.46 (C_quino_).

*1-Hexyl-1,2-dihydro[1,3]thiazolo[3,2-a]quinolin-10-ium bromide* (**11**). ^1^H–NMR (400 MHz, DMSO-*d*_6_): δ 0.80–0.84 (m, 3H, CH_3_), 1.26–1.32 (m, 4H, CH_2_), 1.36–1.51 (m, 2H, CH_2_), 1.59–1.76 (m, 2H, CH_2_), 3.84–3.97 (m, 2H, CH_2_), 4.18–4.22 (m, 1H, SCH_2_), 4.36–4.42 (m, 1H, SCH_2_), 6.21–6.25 (m, 1H, NCH,), 8.12–8.15 (m, 1H, C_quino_), 8.20–8.25 (m, 1H, C_quino_), 8.28–8.32 (m, 2H, C_quino_), 8.32–8.37 (m, 1H, C_quino_), 8.96–8.99 (m, 1H, C_quino_).

^13^C–NMR (101 MHz, DMSO-*d*_6_): δ 24.70 (CH_3_), 26.35, 27.88, 28.38, 29.96, 31.02 (CH_2_), 35.96 (SCH_2_), 68.74 (NCH), 118.70 (C_quino_), 119.05 (C_quino_), 126.30 (C_quino_), 126.73 (C_quino_), 130.52 (C_quino_), 135.06 (C_quino_), 136.76 (C_quino_), 144.84 (C_quino_), 164.30 (C_quino_).

Anal. Calcd for C_17_H_22_NBrS: C 57.95, H 6.29, Br 22.68, N 3.98, S 9.10. Found: C 58.16, H 6.46, Br 23.01, N 4.24, S 9.49.

### 3.3. Synthesis of Compounds ***12**–**15*** by the Reactions of Quinolinesulfenyl Chloride ***2*** and Bromide ***3*** with Cycloalkenes

*8,9,10,10a-Tetrahydro-7aH-cyclopenta[4,5][1,3]thiazolo[3,2-a]quinolin-11-ium chloride* (**12**). A solution of sulfuryl chloride (0.074 g, 0.55 mmol) in methylene chloride (5 mL) was added dropwise to a solution of 2-mercaptoquinoline (0.088 g, 0.55 mmol) in methylene chloride (5 mL), and the mixture was stirred for 15 min at room temperature. The obtained solution of 2-quinolinesulfenyl chloride was added dropwise to a solution of cyclopentene (0.075 g, 1.1 mmol) in methylene chloride (5 mL), and the reaction mixture was stirred for 94 h at room temperature. The solvent was removed by a rotary evaporator. The residue was washed with CCl_4_ and dried in vacuum, giving the product **12** (0.153 g, 81% yield) as a brown oil.

^1^H–NMR (400 MHz, D_2_O): δ 1.83–1.90 (m, 2H, CH_2_), 2.06–2.17 (m, 2H, CH_2_), 2.23–2.31 (m, 1H, CH_2_), 2.47–2.57 (m, 1H, CH_2_), 4.91–4.95 (m, 1H, SCH), 6.03–6.07 (m, 1H, NCH,), 7.62–7.65 (m, 1H, C_quino_), 7.69–7.73 (m, 1H, C_quino_), 7.88–7.90 (m, 1H, C_quino_), 7.94–7.99 (m, 1H, C_quino_), 8.02–8.04 (m, 1H, C_quino_), 8.47–8.50 (m, 1H, C_quino_).

^13^C–NMR (101 MHz, D_2_O): δ 23.77, 34.60, 34.76 (CH_2_), 49.78 (SCH), 75.12 (NCH), 117.67 (C_quino_), 118.06 (C_quino_), 126.76 (C_quino_), 128.28 (C_quino_), 130.42 (C_quino_), 134.90 (C_quino_), 137.50 (C_quino_), 144.69 (C_quino_), 165.39 (C_quino_).

Anal. Calcd for C_14_H_14_NClS: C 63.74, H 5.35, Cl 13.44, N 5.31, S 12.16. Found: C 63.91, H 5.59, Cl 13.79, N 5.52, S 12.52.

*8,9,10,10a-Tetrahydro-7aH-cyclopenta[4,5][1,3]thiazolo[3,2-a]quinoli-11-ium bromide* (**13**). A solution of bromide (0.060 g, 0.38 mmol) in methylene chloride (5 mL) was added dropwise to a solution of 2-mercaptoquinoline (0.061 g, 0.38 mmol) in methylene chloride (5 mL), and the mixture was stirred for 15 min at room temperature. The obtained solution of 2-quinolinesulfenyl chloride was added dropwise to a solution of cyclopentene (0.052 g, 0.76 mmol) in methylene chloride (5 mL), and the reaction mixture was stirred for 20 h at room temperature. The solvent was removed by a rotary evaporator. The residue was washed with CCl_4_ and dried in vacuum, giving product **13** (0.117 g, 100% yield) as a light-yellow oil.

^1^H–NMR (400 MHz, D_2_O): δ 1.76–1.82 (m, 2H, CH_2_), 1.90–1.95 (m, 1H, CH_2_), 2.07–2.12 (m, 1H, CH_2_), 2.19–2.27 (m, 1H, CH_2_), 2.41–2.51 (m, 1H, CH_2_), 4.85–4.89 (m, 1H, SCH), 5.92–5.96 (m, 1H, NCH), 7.55–7.64 (m, 2H, C_quino_), 7.78–7.80 (m, 1H, C_quino_), 7.86–7.94 (m, 2H, C_quino_), 8.38–8.40 (m, 1H, C_quino_).

^13^C–NMR (101 MHz, D_2_O): δ 23.79, 34.67, 34.81 (CH_2_), 49.82 (SCH), 75.02 (NCH), 117.72 (C_quino_), 118.03 (C_quino_), 126.50 (C_quino_), 128.29 (C_quino_), 130.32 (C_quino_), 134.94 (C_quino_), 137.02 (C_quino_), 144.54 (C_quino_), 165.33 (C_quino_).

Anal. Calcd for C_14_H_14_NBrS: C 54.55, H 4.58, Br 28.92, N 4.54, S 10.40. Found: C 54.69, H 4.82, Br 26.34, N 4.71, S 10.86.

*2-(2-Chlorocyclooctylsulfanyl)quinoline* (**14**). A solution of sulfuryl chloride (0.059 g, 0.44 mmol) in methylene chloride (5 mL) was added dropwise to a solution of 2-mercaptoquinoline (0.070 g, 0.44 mmol) in methylene chloride (5 mL), and the mixture was stirred for 15 min at room temperature. A solution of vinyl isobutyl ether (0.098 g, 0.98 mmol) in methylene chloride (5 mL) was added dropwise, and the reaction mixture was stirred for 100 h at room temperature. The solvent was removed by a rotary evaporator. The residue was washed with CCl_4_ and dried in vacuum, giving product **14** (0.119 g, 100% yield) as a light-yellow oil.

^1^H–NMR (400 MHz, acetone-*d_6_*): δ 1.53–1.72 (m, 4H, CH_2_), 1.76–1.87 (m, 3H, CH_2_), 1.93–1.99 (m, 1H, CH_2_), 2.08–2.16 (m, 2H, CH_2_), 2.28–2.41 (m, 2H, CH_2_), 4.51–4.54 (m, 1H, SCH), 4.73–4.78 (m, 1H, CHCl), 7.44–7.45 (m, 1H, C_quino_), 7.55–7.57 (m, 1H, C_quino_), 7.75–7.79 (m, 1H, C_quino_), 7.92–7.94 (m, 1H, C_quino_), 8.09–8.13 (m, 1H, C_quino_), 8.25–8.27 (m, 1H, C_quino_).

^13^C–NMR (101 MHz, acetone-*d_6_*): δ 24.10 (CH_2_), 26.00 (CH_2_), 26.80 (CH_2_), 27.87 (CH_2_), 32.14 (CH_2_), 32.73 (CH_2_), 53.10 (SCH), 66.44 (CHCl), 122.16 (C_quino_), 127.03 (C_quino_), 128.02 (C_quino_), 128.99 (C_quino_), 130.77 (C_quino_), 131.17 (C_quino_), 131.64 (C_quino_), 138.50 (C_quino_), 159.57 (C_quino_).

Anal. Calcd for C_17_H_20_NClS: C 66.76, H 6.59, Cl 11.59, N 4.58, S 10.48. Found: C 66.93, H 6.71, Cl 11.93, N 4.79, S 12.00.

*7a,8,9,10,11,12,13,13a-Octahydrocyclocta[4,5][1,3]thiazolo[3,2-a]quinolin-14-ium bromide* (**15**). A solution of bromide (0.074 g, 0.46 mmol) in methylene chloride (5 mL) was added dropwise to a solution of 2-mercaptoquinoline (0.075 g, 0.46 mmol) in methylene chloride (5 mL), and the mixture was stirred for 15 min at room temperature. The obtained solution of 2-quinolinesulfenyl bromide was added dropwise to a solution of cyclooctene (0.102 g, 0.92 mmol) in methylene chloride (5 mL), and the reaction mixture was stirred for 20 h at room temperature. The solvent was removed by a rotary evaporator. The residue was washed with CCl_4_ and dried in vacuum, giving product **15** (0.120 g, 77% yield) as a light-yellow oil.

^1^H–NMR (400 MHz, D_2_O): δ 1.24–1.39 (m, 3H, CH_2_), 1.59–1.71 (m, 3H, CH_2_), 1.81–1.91 (m, 3H, CH_2_), 1.99–2.08 (m, 1H, CH_2_), 2.11–2.25 (m, 1H, CH_2_), 2.35–2.44 (m, 1H, CH_2_), 4.61–4.67 (m 1H, SCH), 5.87–5.92 (m, 1H, NCH), 7.66–7.72 (m, 2H, C_quino_), 7.80–7.83 (m, 1H, C_quino_), 7.96–8.01 (m, 1H, C_quino_), 8.03–8.06 (m, 1H, C_quino_), 8.50–8.52 (m, 1H, C_quino_).

^13^C–NMR (101 MHz, D_2_O): δ 23.20, 23.76, 24.10, 25.66, 26.11, 27.75 (CH_2_), 50.93 (SCH), 71.74 (NCH), 117.64 (C_quino_), 117.72 (C_quino_), 126.78 (C_quino_), 127.96 (C_quino_), 130.37 (C_quino_), 134.98 (C_quino_), 136.63 (C_quino_), 144.54 (C_quino_), 162.63 (C_quino_).

Anal. Calcd for C_17_H_20_NBrS: C 58.28, H 5.75, Br 22.81, N 4.00, S 9.15. Found: C 58.61, H 6.02, Br 23.14, N 4.31, S 9.71.

### 3.4. Synthesis of Compounds ***16*** and ***17*** by the Reactions of Quinolinesulfenyl Chloride ***2*** with Styrene and Isoeugenol

*1-Phenyl-1,2-dihydro[1,3]thiazolo[3,2-a]quinolin-10-ium chloride* (**16**). A solution of sulfuryl chloride (0.047 g, 0.34 mmol) in methylene chloride (5 mL) was added dropwise to a solution of 2-mercaptoquinoline (0.056 g, 0.34 mmol) in methylene chloride (5 mL), and the mixture was stirred for 15 min at room temperature. The obtained solution of 2-quinolinesulfenyl chloride was added dropwise to a solution of styrene (0.072 g, 0.68 mmol) in methylene chloride (5 mL), and the reaction mixture was stirred for 20 h at room temperature. The solvent was removed by a rotary evaporator. The residue was washed with CCl_4_ and dried in vacuum, giving product **16** (0.104 g, 100% yield) as a yellow oil.

^1^H–NMR (400 MHz, D_2_O): δ 3.50–3.54 (m, 1H, SCH_2_), 4.31–4.36 (m, 1H, SCH_2_), 6.89–6.95 (m, 2H, Ar), 6.93–6.97 (m, H, NCH), 7.04–7.12 (m, 3H, Ar), 7.40–7.44 (m, 1H, C_quino_), 7.49–7.56 (m, 2H, C_quino_), 7.78–7.82 (m, 1H, C_quino_), 7.85–7.90 (m, 1H, C_quino_), 8.52–8.56 (m, 1H, C_quino_).

^13^C–NMR (101 MHz, D_2_O): δ 37.87 (SCH_2_), 71.06 (NCH), 118.10 (C_quino_), 118.30 (C_quino_), 125.39 (Ar), 126.73 (C_quino_), 128.42 (C_quino_), 129.51 (Ar), 129.65 (Ar), 130.47 (C_quino_), 135.07 (C_quino_), 135.21 (C_quino_), 137.12 (Ar), 145.88 (C_quino_), 166.15 (C_quino_).

Anal. Calcd for C_17_H_14_NClOS: C 68.10, H 4.71, Cl 11.82, N 4.67, S 10.70. Found: C 68.34, H 4.97, Cl 12.12, N 4.81, S 11.20.

*trans-1-(Hydroxy-3-methoxyphenyl)-2-methyl-1,2-dihydro[1,3]thiazolo[3,2-a]quinolin-10-ium chloride* (**17**). A solution of sulfuryl chloride (0.078 g, 0.57 mmol) in methylene chloride (5 mL) was added dropwise to a solution of 2-mercaptoquinoline (0.093 g, 0.57 mmol) in methylene chloride (5 mL), and the mixture was stirred for 15 min at room temperature. The obtained solution of 2-quinolinesulfenyl chloride was added dropwise to a solution of isoeugenol (0.189 g, 1.15 mmol) in methylene chloride (5 mL), and the reaction mixture was stirred for 20 h at room temperature. After filtration, the solvent was removed by a rotary evaporator, and the residue was washed with CCl_4_ and dried in vacuum, giving product **17** (0.166 g, 80% yield) as an orange oil.

^1^H–NMR (400 MHz, D_2_O): δ 1.70 (d, *J* 7.0 Hz, 3H, CH_3_), 3.76 (s, 3H, O;CH_3_), 4.07–4.12 (m, 1H, SCH), 6.21 (d, *J* 8.2 Hz, 1H, NCH), 6.56–6.60 (m, 1H, Ar), 6.66 (s, 1H, Ar), 6.91 (s, 1H, Ar), 7.62–7.66 (m, 1H, C_quino_), 7.74–7.79 (m, 2H, C_quino_), 7.93–7.96 (m, 1H, C_quino_), 8.08–8.11 (m, 1H, C_quino_), 8.72–8.75 (m, 1H, C_quino_).

^13^C–NMR (101 MHz, D_2_O): δ 22.38 (CH_3_), 50.80 (SCH), 55.77 (OCH_3_), 77.37 (NCH), 109.61 (Ar), 115.50 (Ar), 117.48 (Ar), 118.08 (C_quino_), 118.51 (C_quino_), 126.83 (C_quino_), 126.94 (Ar), 128.42 (C_quino_), 130.42 (C_quino_), 134.97 (C_quino_), 137.59 (C_quino_), 145.78 (Ar), 145.86 (C_quino_), 148.07 (Ar), 164.96 (C_quino_).

Anal. Calcd for C_19_H_18_NClO_2_S: C 63.41, H 5.04, Cl 9.85, N 3.89, S 8.91. Found: C 63.63, H 5.21, Cl 10.17, N 4.00, S 9.27.

## 4. Conclusions

Novel reagents 2-quinolinesulfenyl chloride and bromide have been involved in the preparation of condensed heterocyclic sulfur–nitrogen compounds. The simple and efficient method for generation of 2-quinolinesulfenyl chloride and bromide is based on the action of sulfuryl chloride or bromine on available 2-mercaptoquinoline. Regioselective synthesis of novel 2-alkyl-1,2-dihydro[1,3]thiazolo[3,2-*a*]quinolin-10-ium derivatives in 95–100% yields has been developed by annulation reactions of 2-quinolinesulfenyl chloride and bromide with 1-alkenes. Condensed tetracyclic products have been obtained by the reactions of 2-quinolinesulfenyl chloride and bromide and cycloalkenes.

The reactions of cyclopentene with both 2-quinolinesulfenyl chloride and bromide at room temperature afforded condensed tetracyclic products in high yields. The reaction of 2-quinolinesulfenyl chloride with cyclooctene led to an electrophilic addition product in quantitative yield. When 2-quinolinesulfenyl bromide was involved in the reaction with cyclooctene under similar conditions, the tetracyclic condensed compound was obtained in quantitative yield.

The reactions of 2-quinolinesulfenyl chloride with styrene and natural compound isoeugenol proceeded in a regioselective manner but with opposite regiochemistry compared to the reactions of 2-quinolinesulfenyl chloride with terminal alkenes. The electrophilic addition of the sulfur atom of 2-quinolinesulfenyl chloride occurred to the β-carbon atom of the double bond of styrene and isoeugenol in a Markovnikov fashion.

The reaction of 2-quinolinesulfenyl chloride with terminal alkenes was supposed to proceed via intermediate thiiranium cation, and nucleophilic attack of the nitrogen atom of the quinoline ring occurred at the least substituted carbon atom of thiiranium ion, leading to the formation of condensed products in an anti-Markovnikov fashion.

The obtained condensed sulfur–nitrogen heterocycles are novel water-soluble functionalized compounds with potential biological activity.

## Data Availability

Data is available in this article and Supplementary Materials.

## Supplementary Materials

The following are available online. Supporting Information file: examples of ^1^H- and ^13^C-NMR spectra of the obtained compounds.

## Sample Availability

Samples of the compounds are not available from the authors.
