# Supplementary material for: Efficient Regioselective Synthesis of Novel Condensed Sulfur–Nitrogen Heterocyclic Compounds Based on Annulation Reactions of 2-Quinolinesulfenyl Halides with Alkenes and Cycloalkenes
Source: Molecules. 2021 Aug 10;26(16):4844. doi: 10.3390/molecules26164844 (PMC8399591; doi:10.3390/molecules26164844)
Supplement: Supplementary file 1 [file molecules-26-04844-s001.zip › molecules-1301940-supplementary.pdf]

Supporting information

Efficient Regioselective Synthesis of Novel Condensed Sulfur-Nitrogen Heterocyclic Compounds Based on Annulation Reactions of 2-Quinolinesulfenyl Halides with Alkenes and Cycloalkenes

Vladimir A. Potapov,*, Roman S. Ishigeev and Svetlana V. Amosova

A. E. Favorsky Irkutsk Institute of Chemistry, Siberian Division of The Russian Academy of Sciences, 1 Favorsky Str., Irkutsk 664033, Russian Federation; v.a.potapov@mail.ru

Table of Contents

Experimental (General Information) 2

Examples of NMR Spectra 3-12

Experimental (General Information)

^{1}H (400.1 MHz) and ^{13}C (100.6 MHz) NMR spectra were recorded on a Bruker DPX-400 spectrometer (Bruker BioSpin GmbH, Rheinstetten, Germany) in 2-5% solution in D_2O. ^{1}H and ^{13}C chemical shifts (δ) are reported in parts per million (ppm), relative to tetramethylsilane (external) or to the residual solvent peaks of D_2O (δ = 4.79), acetone-d_6 (δ = 2.05 and 29.84 ppm) and DMSO-d_6 (δ = 2.50 and 39.52 ppm for ^{1}H and ^{13}C NMR, respectively). Elemental analysis was performed on a Thermo Scientific FLASH 2000 Organic Elemental Analyzer (Thermo Fisher Scientific Inc., Milan, Italy). Melting points were determined on a Kofler Hot-Stage Microscope PolyTherm A apparatus (Wagner & Munz GmbH, München, Germany). Absolute solvents were used in the reactions.

Examples of ^{1}H and ^{13}C NMR spectra

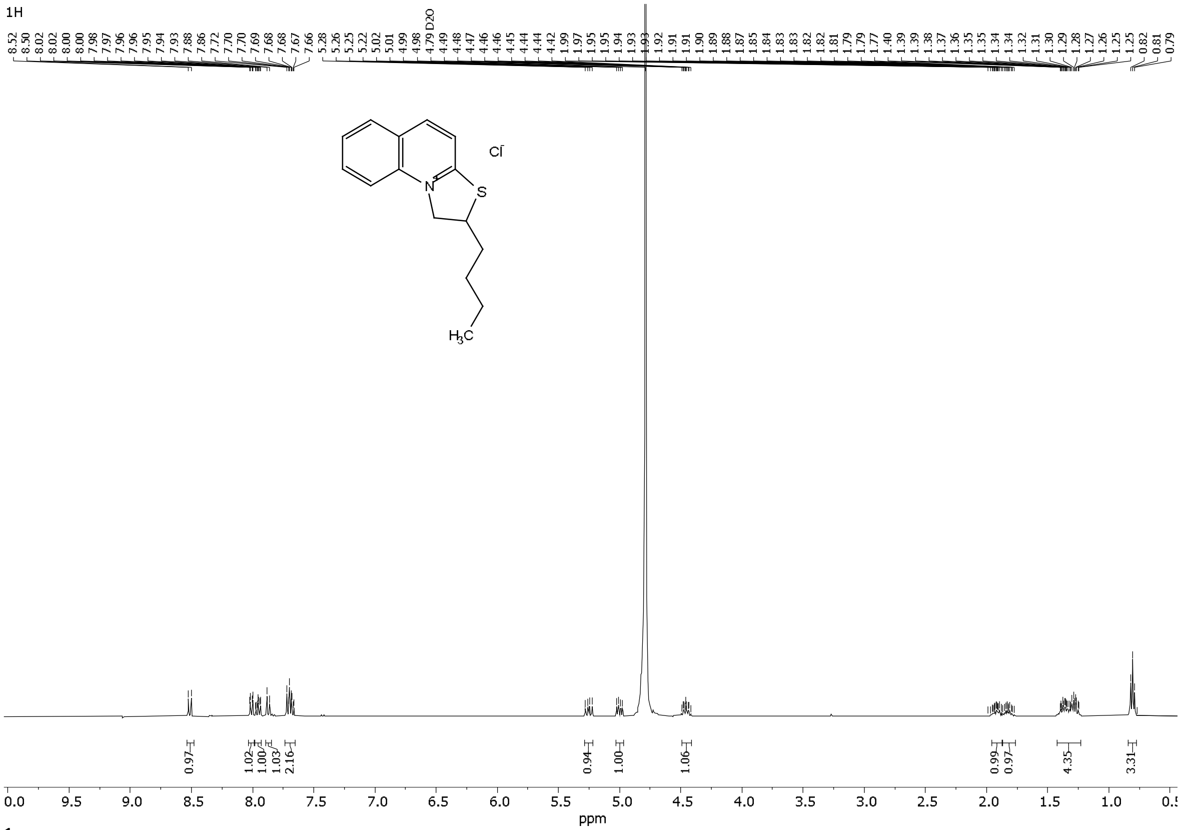

^{1}H-NMR (D_2O) spectrum of 2-butyl-1,2-dihydro[1,3]thiazolo[3,2-*a*]quinolin-10-ium chloride (4)

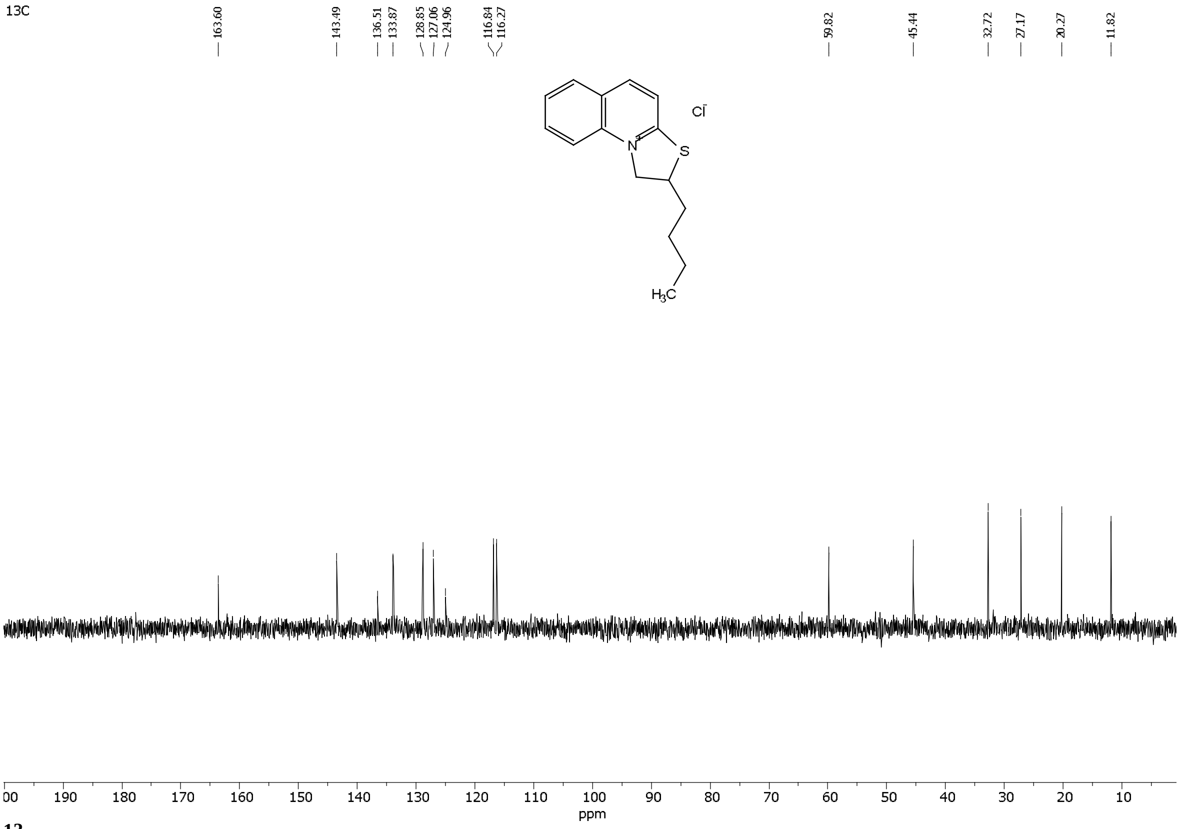

^{13}C-NMR (D_2O) spectrum of 2-butyl-1,2-dihydro[1,3]thiazolo[3,2-*a*]quinolin-10-ium chloride (4)

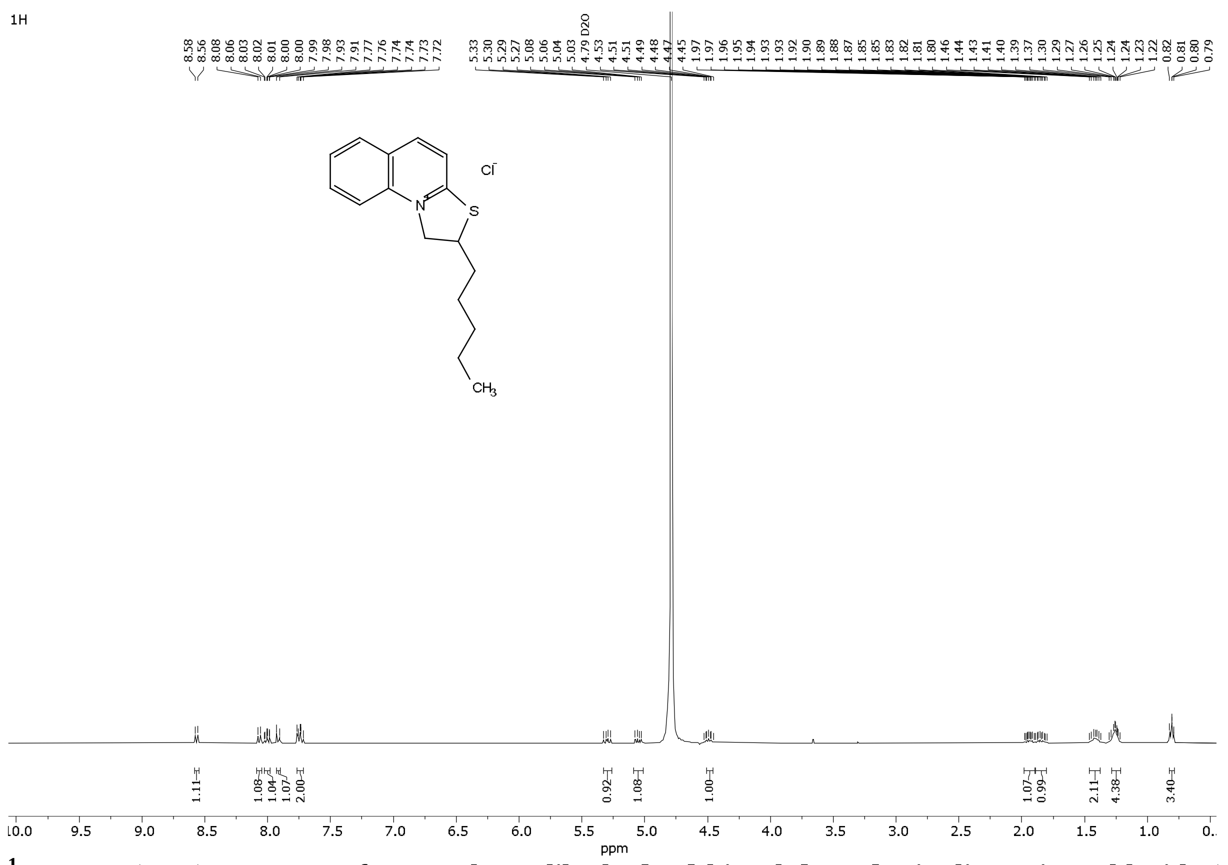

^{1}H-NMR (D_2O) spectrum of 2-pentyl-1,2-dihydro[1,3]thiazolo[3,2-*a*]quinolin-10-ium chloride (5)

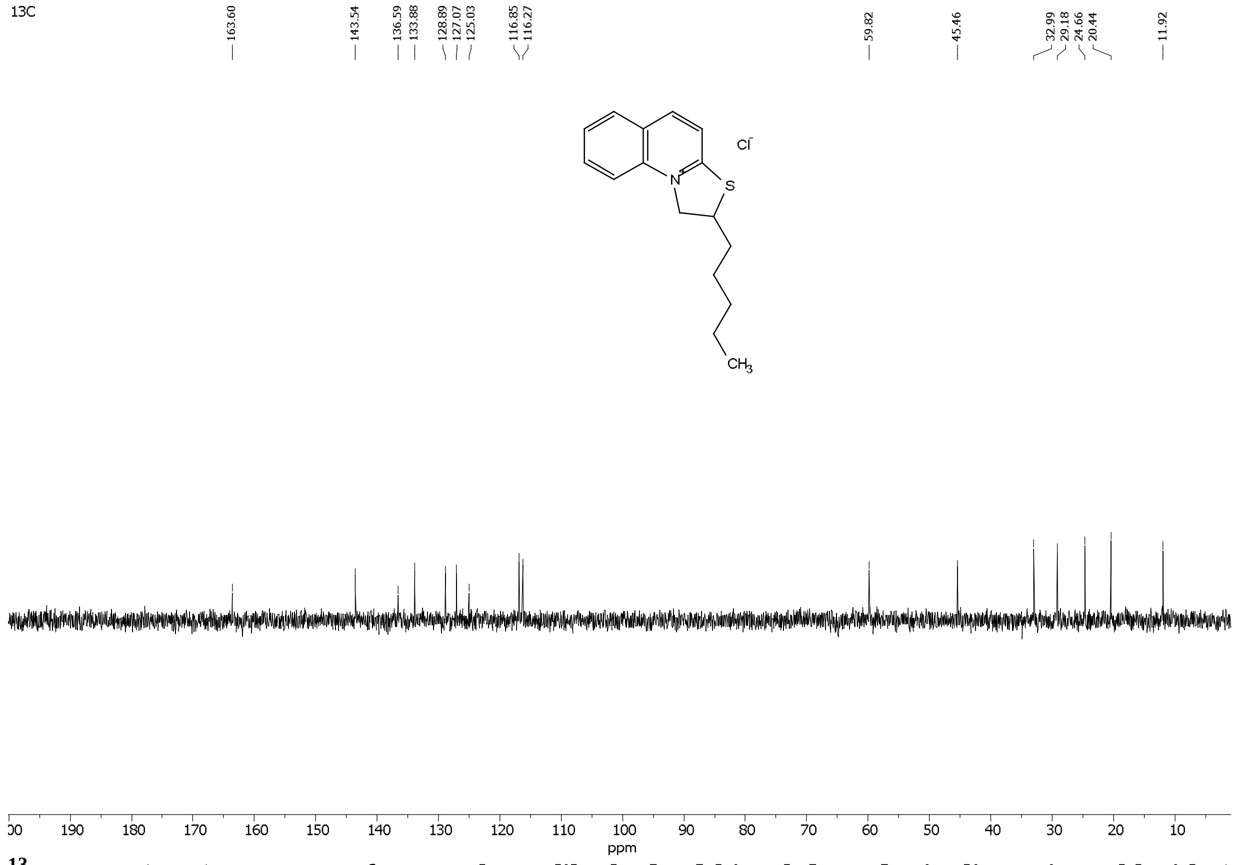

^{13}C-NMR (D_2O) spectrum of 2-pentyl-1,2-dihydro[1,3]thiazolo[3,2-*a*]quinolin-10-ium chloride (5)

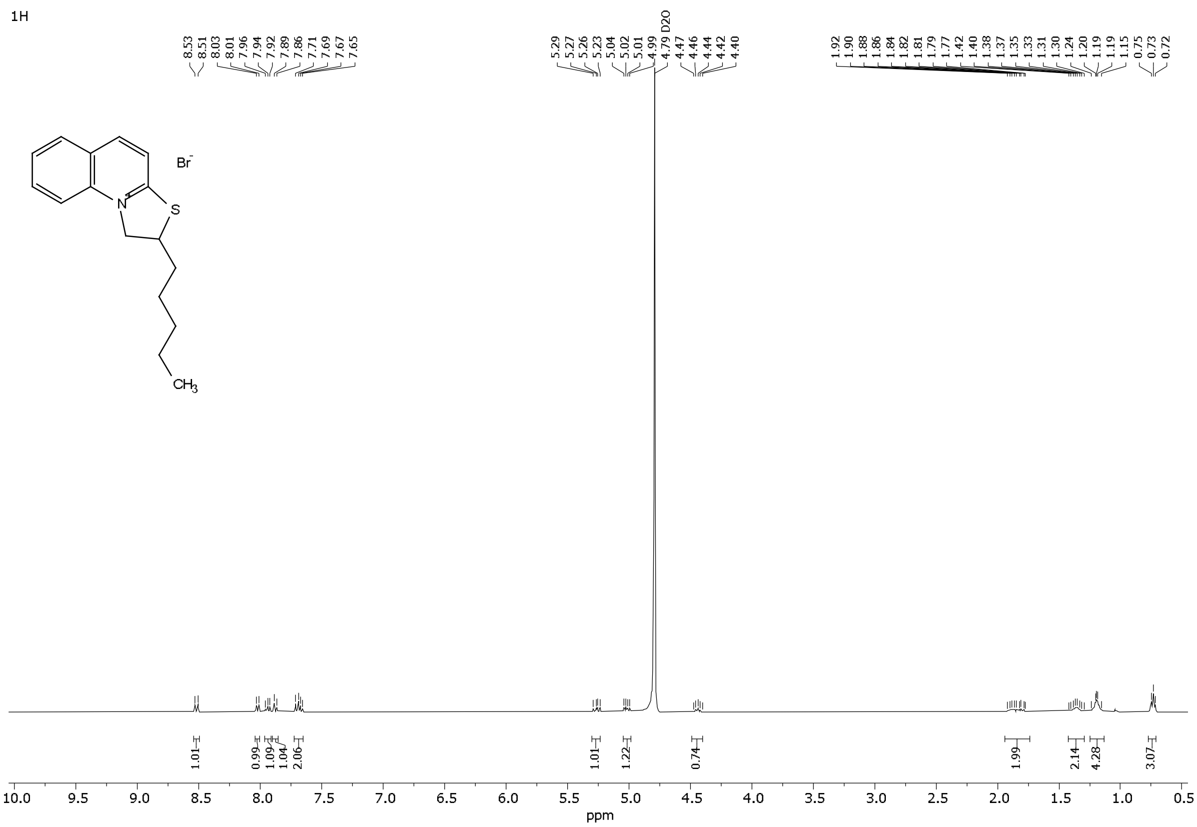

^{1}H-NMR (D_2O) spectrum of 2-pentyl-1,2-dihydro[1,3]thiazolo[3,2-*α*]quinolin-10-ium bromide (7)

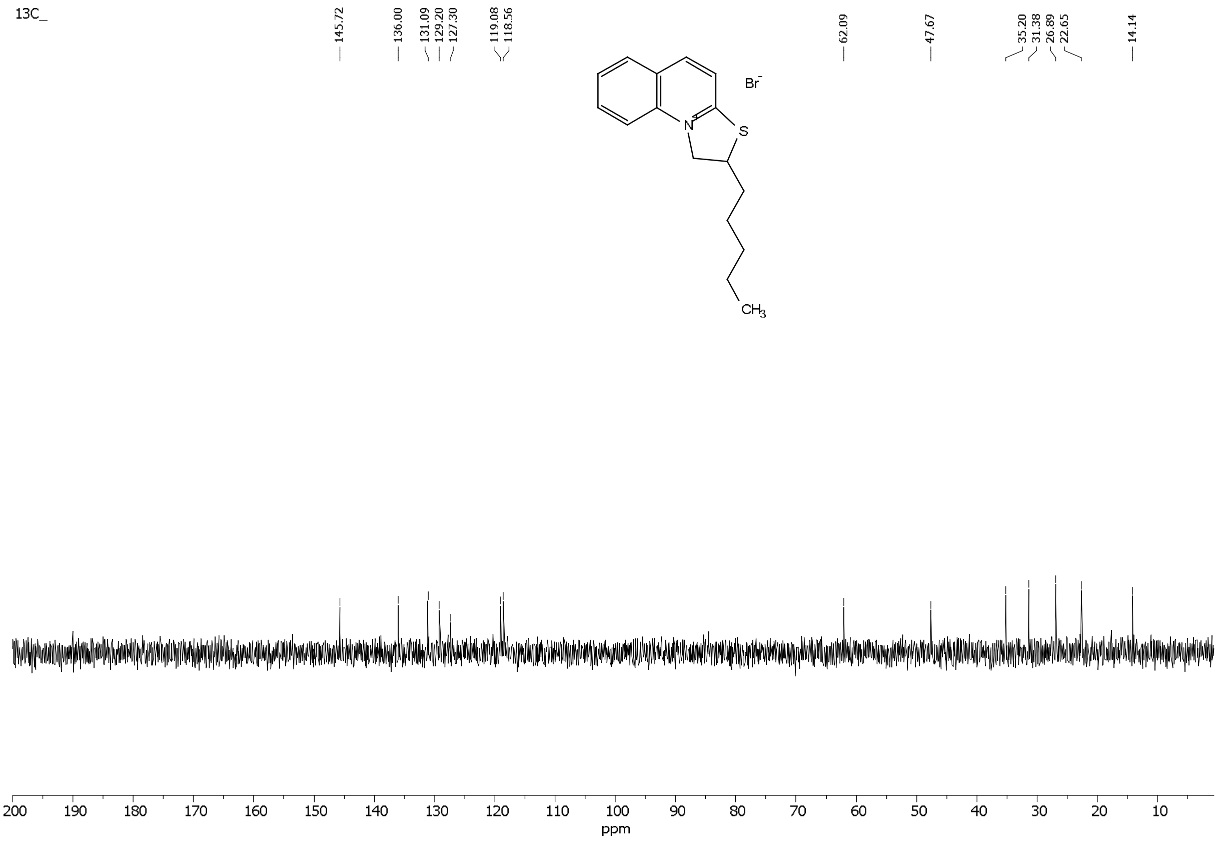

^{13}C-NMR (D_2O) spectrum of 2-pentyl-1,2-dihydro[1,3]thiazolo[3,2-*a*]quinolin-10-ium bromide (7)

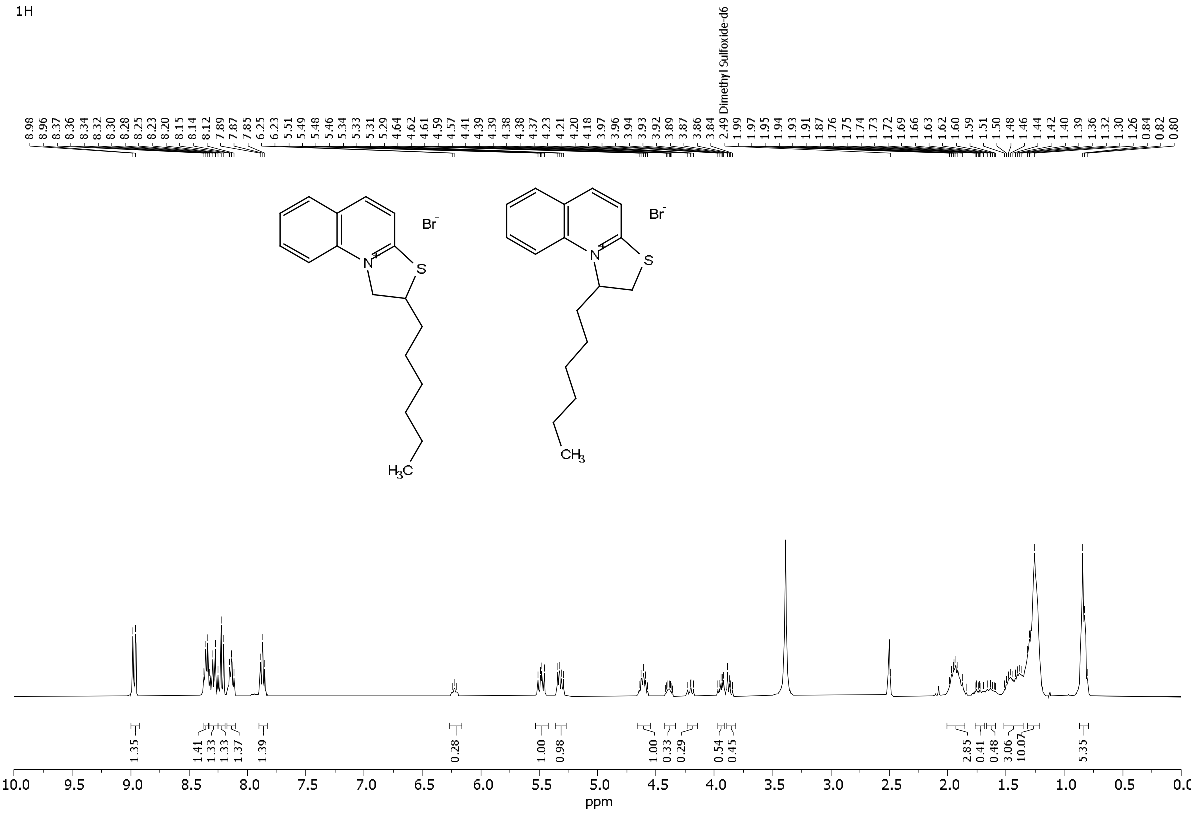

^{1}H-NMR (DMSO-d_6) spectrum of 2-hexyl-1,2-dihydro[1,3]thiazolo[3,2-*a*]quinolin-10-ium bromide (9) and 1-hexyl-1,2-dihydro[1,3]thiazolo[3,2-*a*]quinolin-10-ium bromide (11)

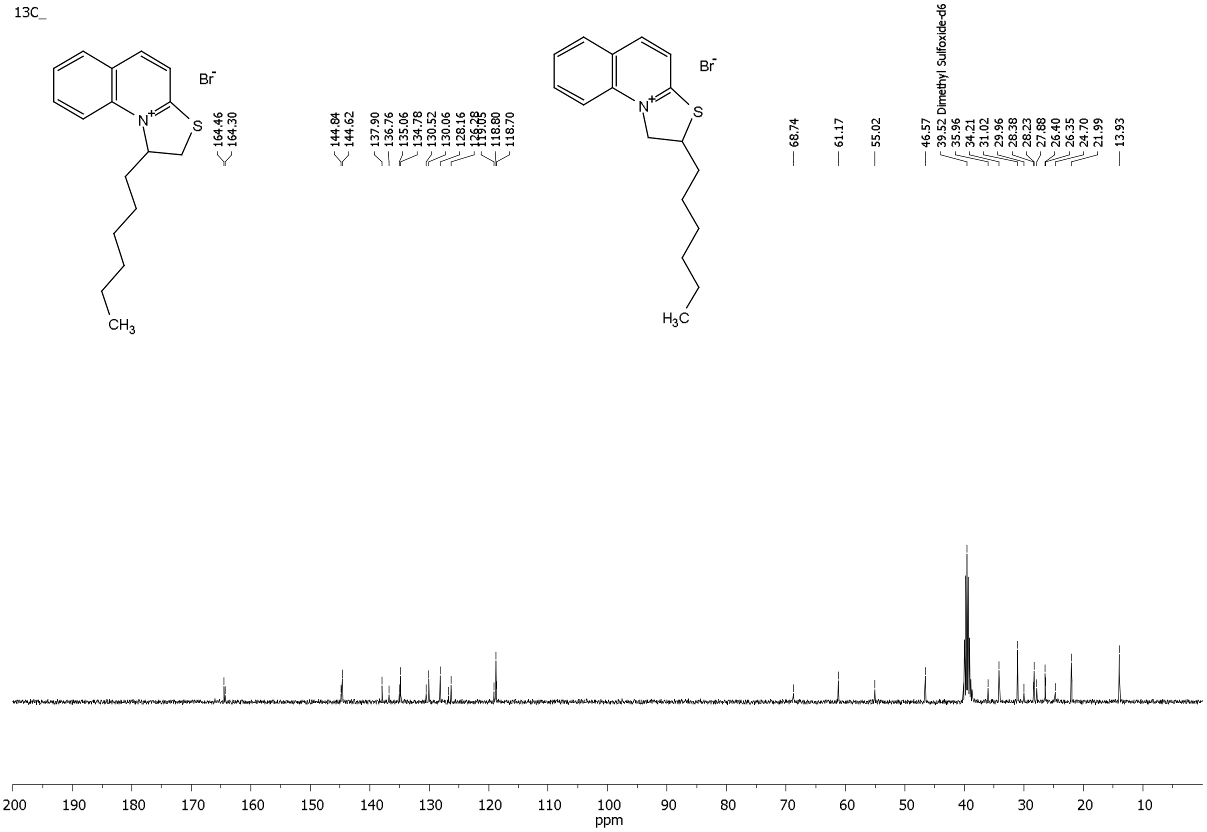

^{13}C-NMR (DMSO-d_6) spectrum of 2-hexyl-1,2-dihydro[1,3]thiazolo[3,2-*a*]quinolin-10-ium bromide (9) and 1-hexyl-1,2-dihydro[1,3]thiazolo[3,2-*a*]quinolin-10-ium bromide (11)

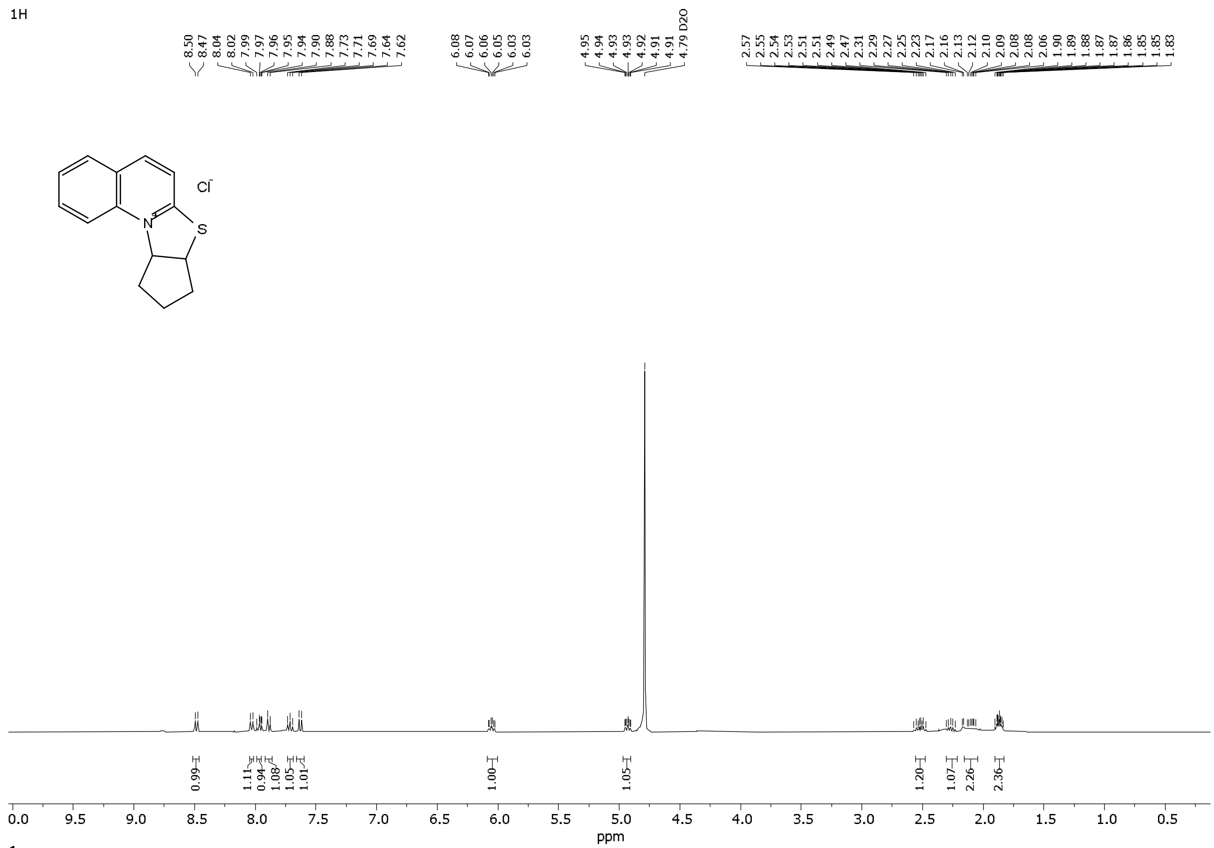

^{1}H-NMR (D_2O) spectrum of 8,9,10,10a-Tetrahydro-7a*H*-cyclopenta[4,5][1,3]thiazolo[3,2-*a*]quinolin-11-ium chloride (12)

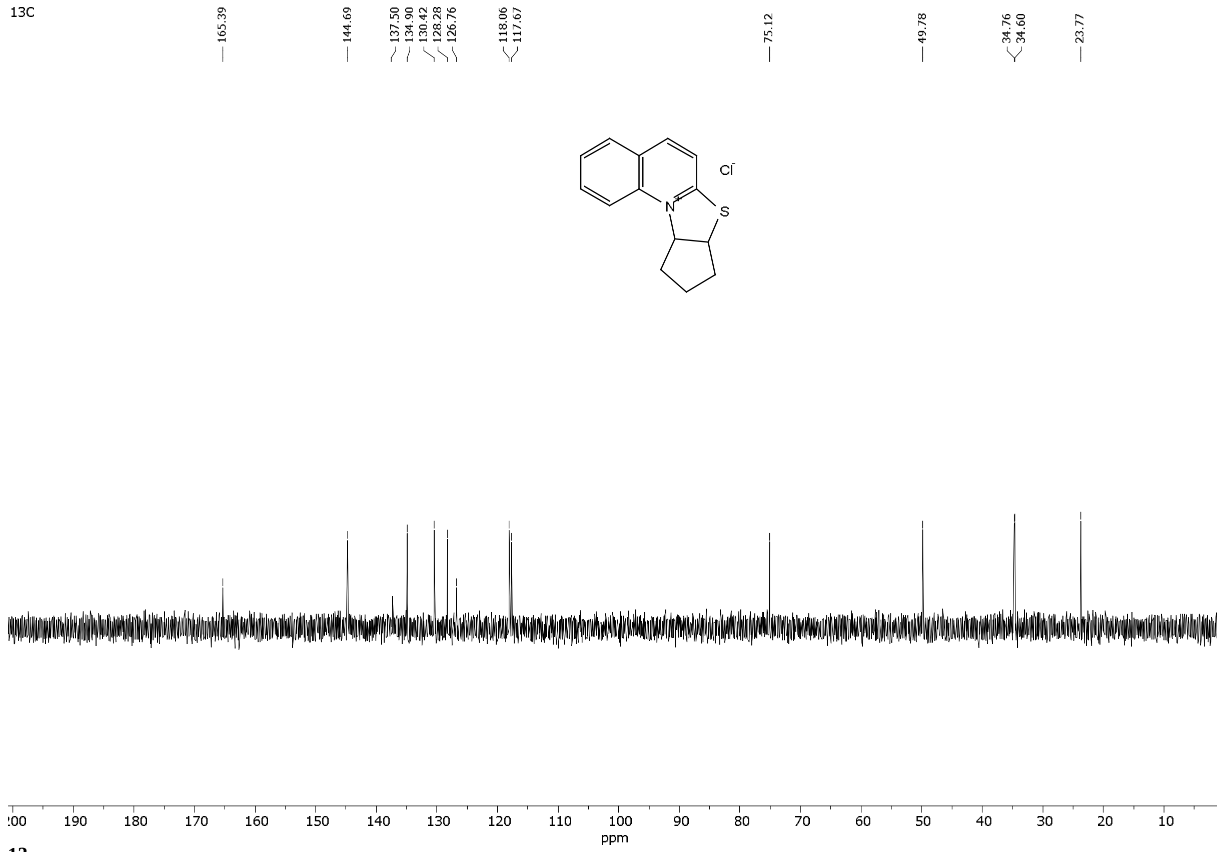

^{13}C-NMR (D_2O) spectrum of 8,9,10,10a-Tetrahydro-7a*H*-cyclopenta[4,5][1,3]thiazolo[3,2-*a*]quinolin-11-ium chloride (12)

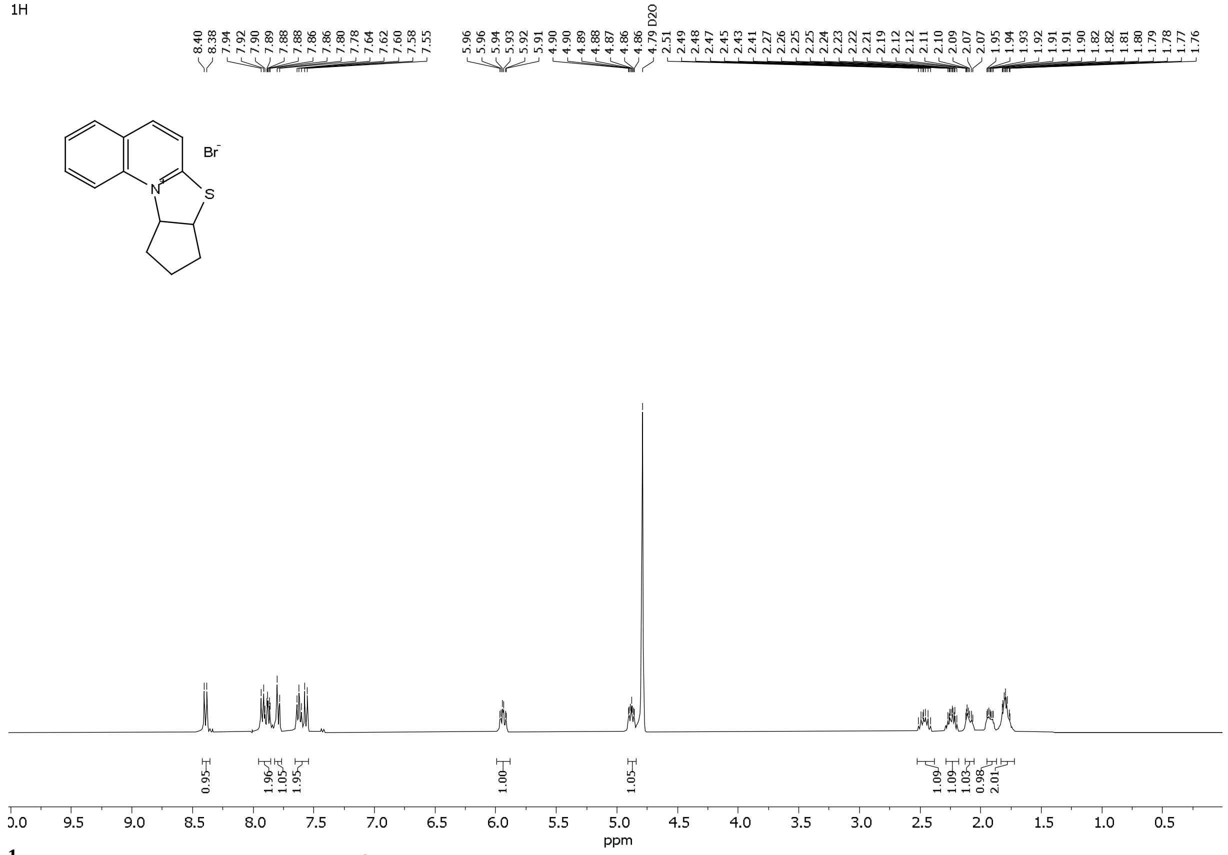

^{1}H-NMR (D_2O) spectrum of 8,9,10,10a-Tetrahydro-7a*H*-cyclopenta[4,5][1,3]thiazolo[3,2-*a*]quinolin-11-ium bromide (13)

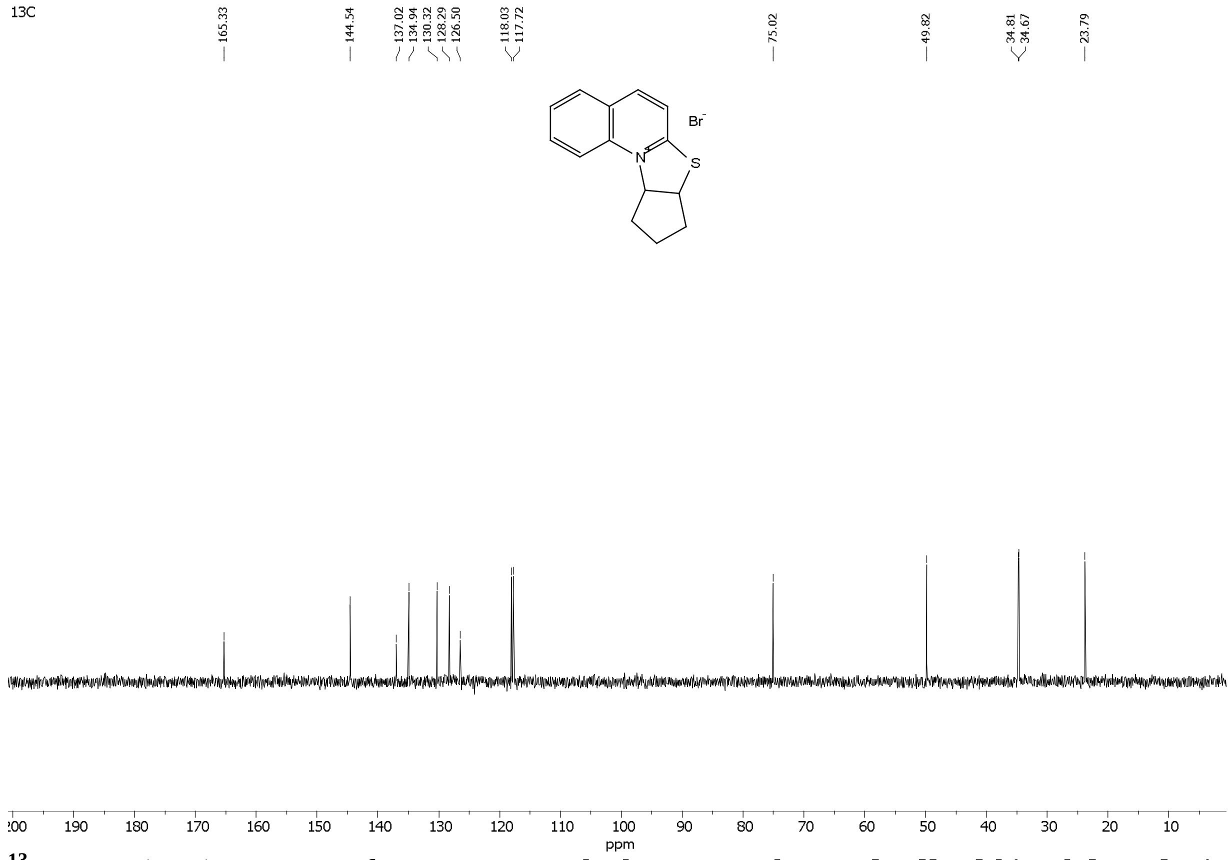

^{13}C-NMR (D_2O) spectrum of 8,9,10,10a-tetrahydro-7a*H*-cyclopenta[4,5][1,3]thiazolo[3,2-*a*]quinolin-11-ium bromide (13)

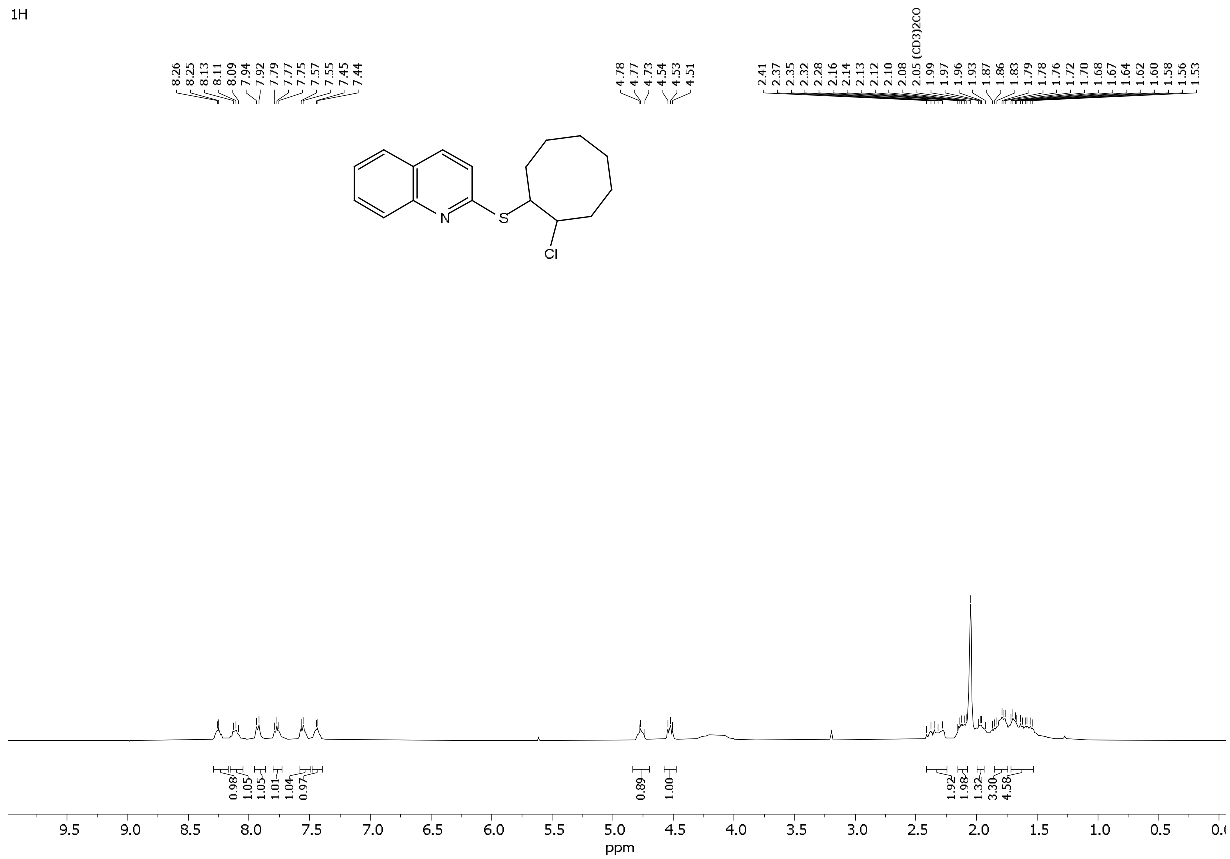

^{1}**H-NMR (acetone-*d*$_6$) spectrum of** 2-[(2-chlorocyclooctyl)sulfanyl]quinoline (14)

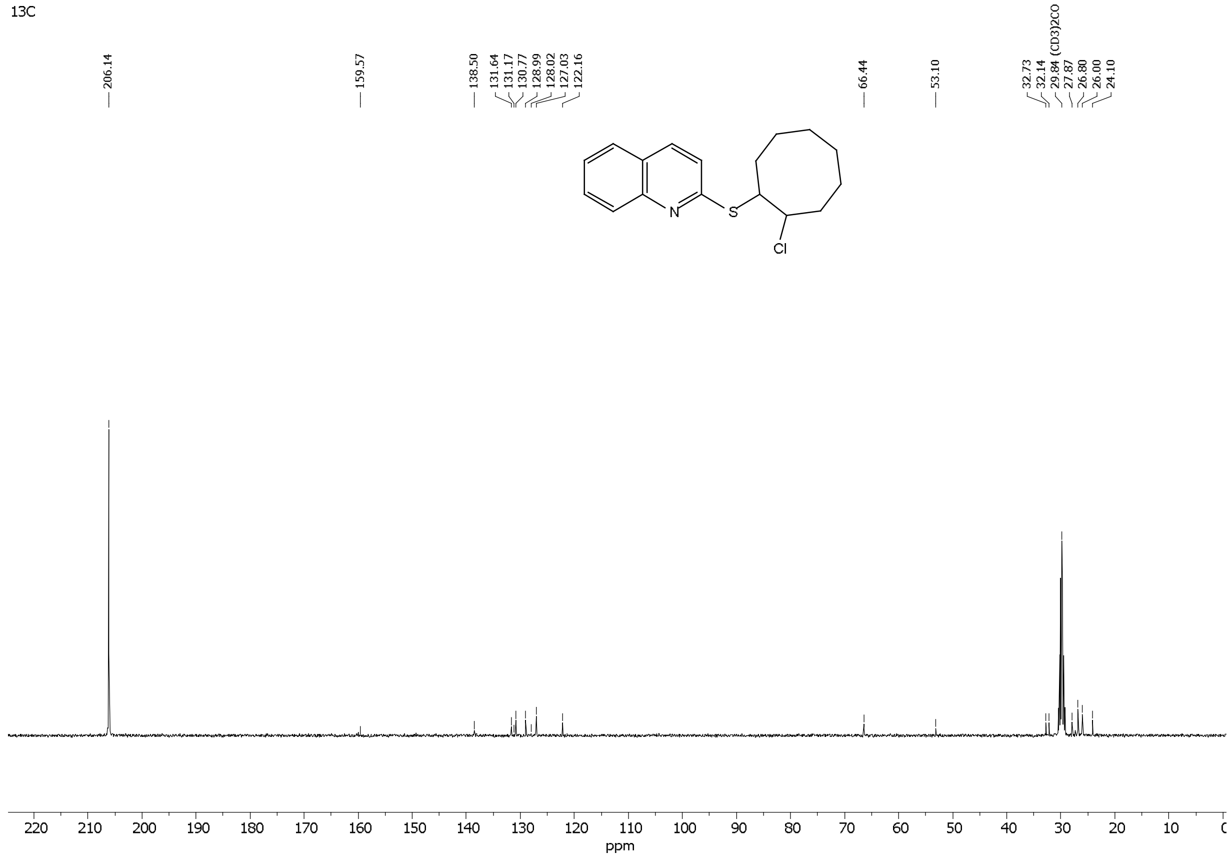

^{13}**C-NMR (acetone-*d*$_6$) spectrum of** 2-[(2-chlorocyclooctyl)sulfanyl]quinoline (14)

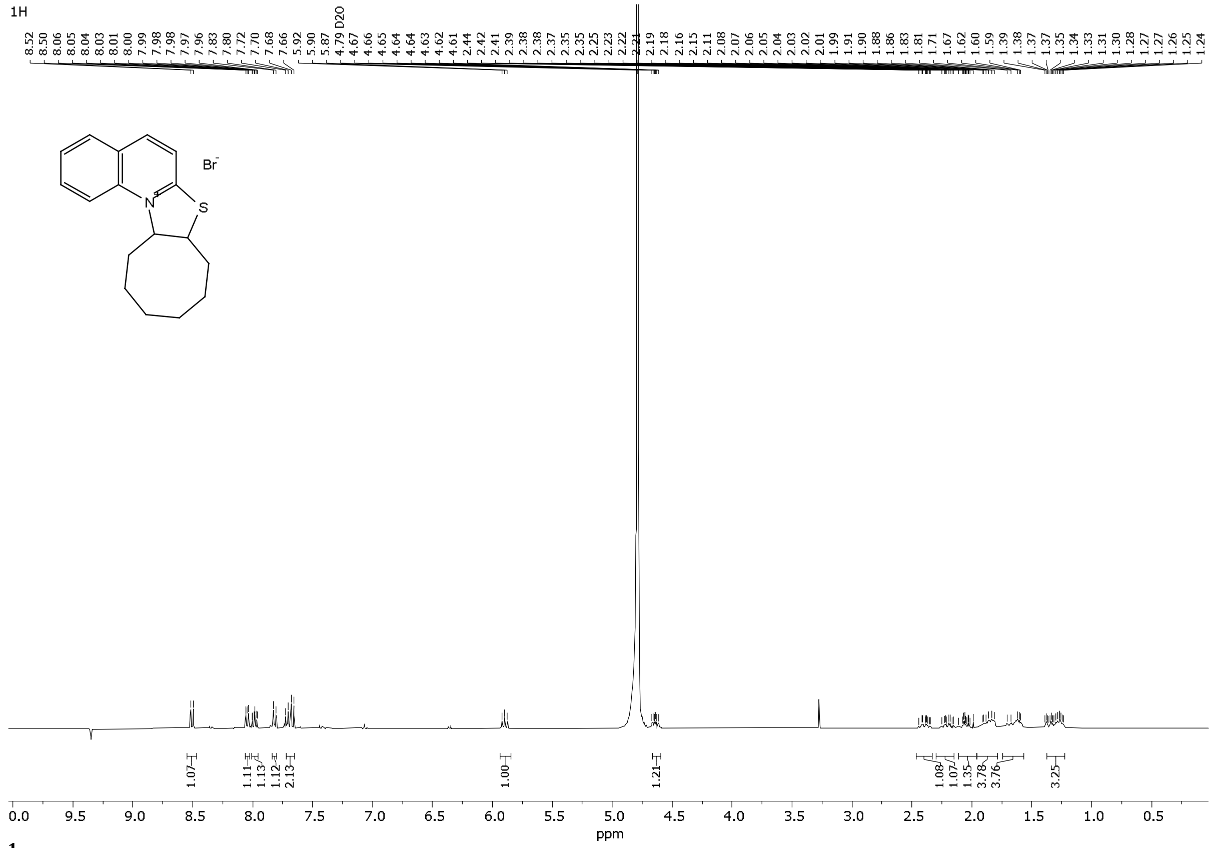

^{1}H-NMR (D_2O) spectrum of 7a,8,9,10,11,12,13,13a-Octahydrocycloocta[4,5][1,3]thiazolo[3,2-*a*]quinolin-14-ium bromide (15)

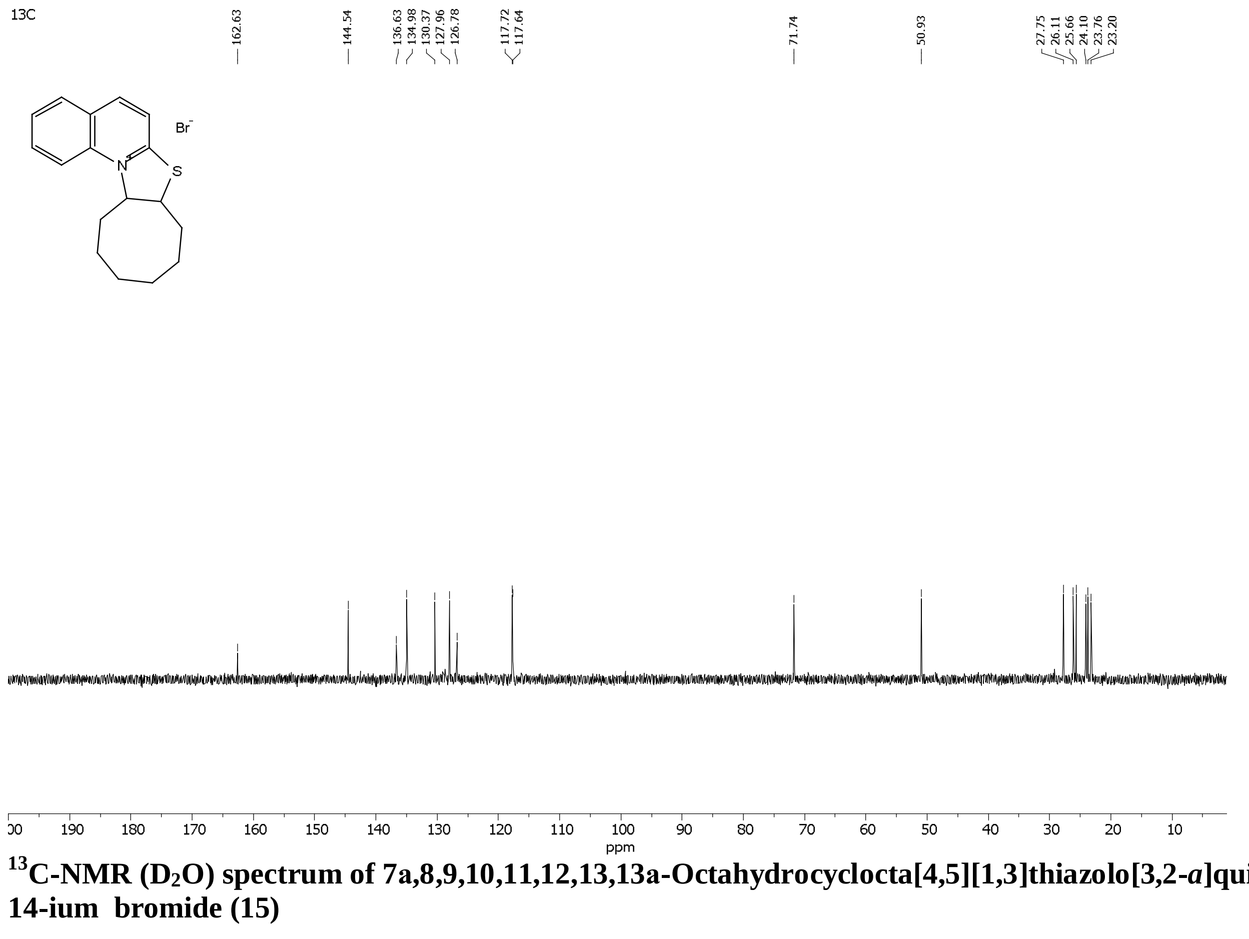

^{13}C-NMR (D_2O) spectrum of 7a,8,9,10,11,12,13,13a-Octahydrocycloocta[4,5][1,3]thiazolo[3,2-*a*]quinolin-14-ium bromide (15)

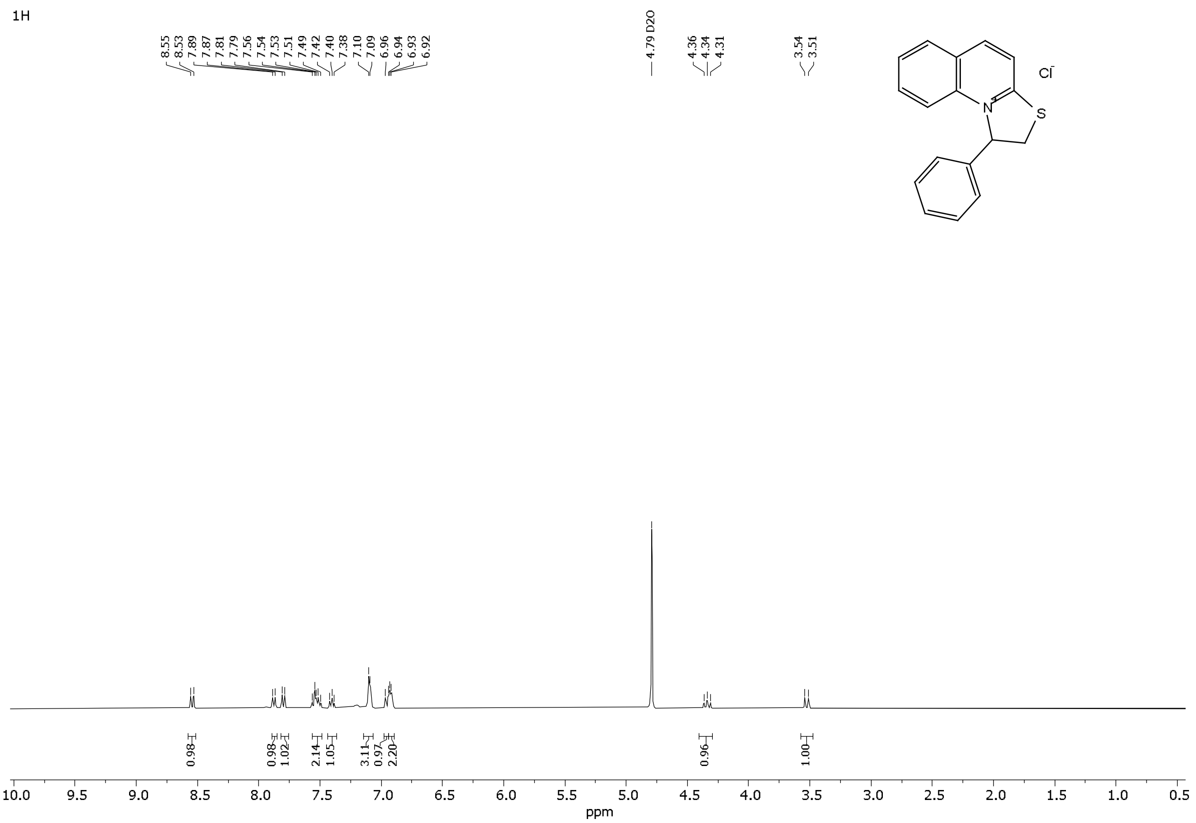

^{1}H-NMR (D_2O) spectrum of 1-phenyl-1,2-dihydro[1,3]thiazolo[3,2-*a*]quinolin-10-ium chloride (16)

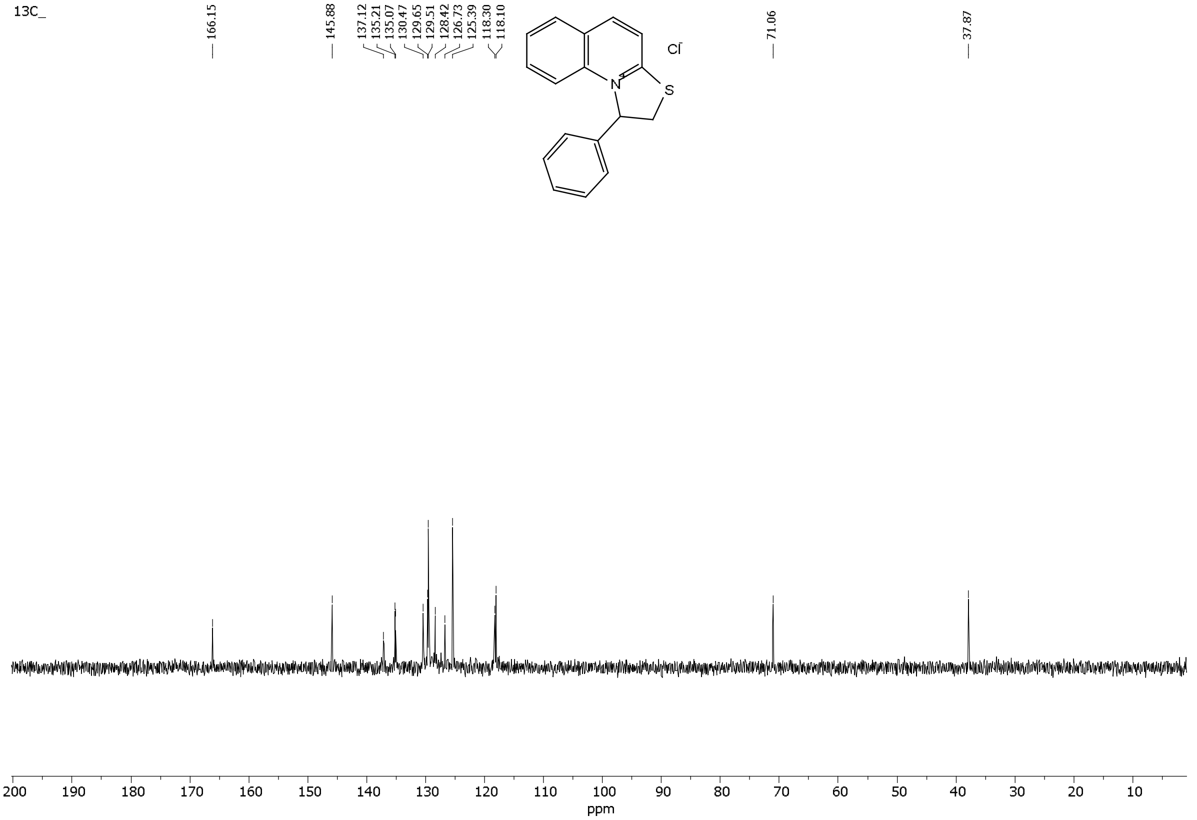

^{13}C-NMR (D_2O) spectrum of 1-phenyl-1,2-dihydro[1,3]thiazolo[3,2-*a*]quinolin-10-ium chloride (16)

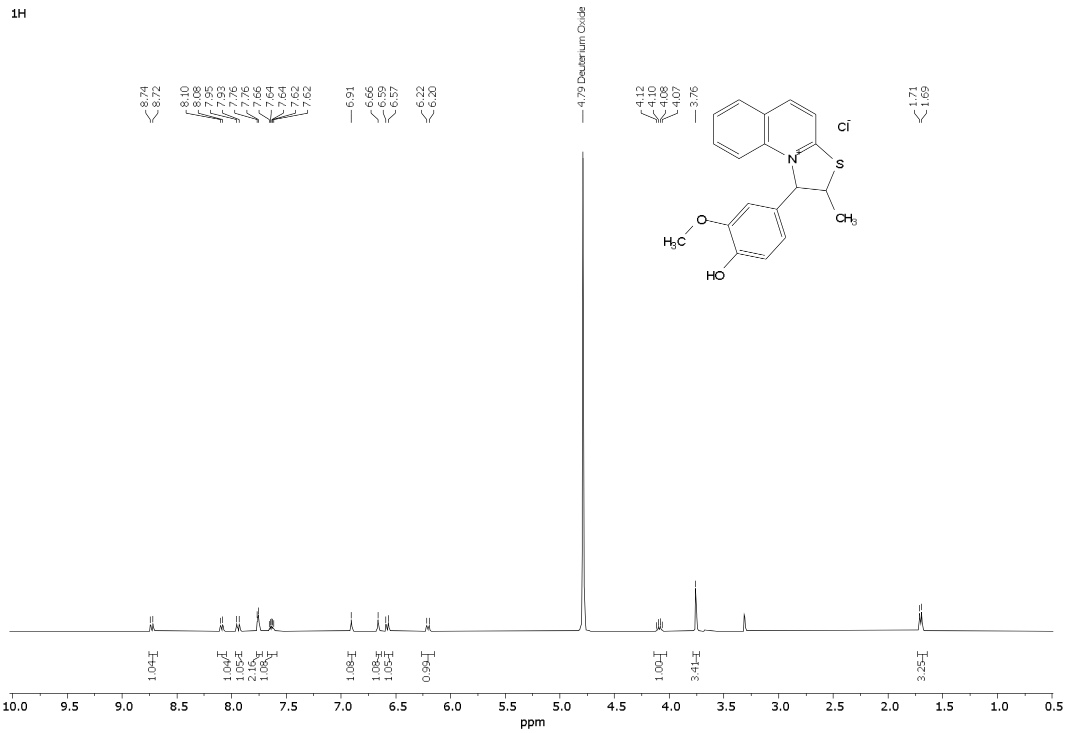

^{1}H-NMR (D_2O) spectrum of 1-(4-hydroxy-3-methoxyphenyl)-2-methyl-1,2-dihydro[1,3]thiazolo[3,2-*a*]quinolin-10-ium chloride

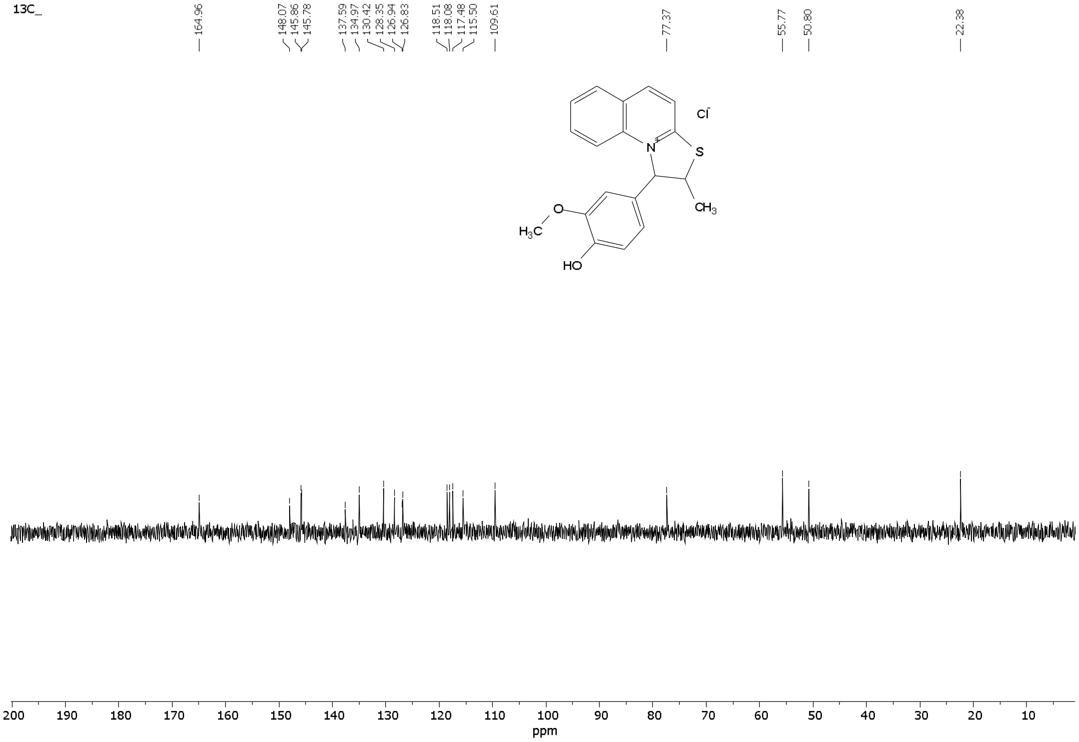

^{13}C-NMR (D_2O) spectrum of 1-(4-hydroxy-3-methoxyphenyl)-2-methyl-1,2-dihydro[1,3]thiazolo[3,2-*a*]quinolin-10-ium chloride (17)
